# Elucidation of the late steps in the glycan‐dependent ERAD of soluble misfolded glycoproteins

**DOI:** 10.1111/tpj.17185

**Published:** 2024-12-06

**Authors:** Jennifer Schoberer, Ulrike Vavra, Yun‐Ji Shin, Clemens Grünwald‐Gruber, Richard Strasser

**Affiliations:** ^1^ Department of Applied Genetics and Cell Biology, Institute of Plant Biotechnology and Cell Biology University of Natural Resources and Life Sciences Muthgasse 18 Vienna A‐1190 Austria; ^2^ Core Facility Mass Spectrometry University of Natural Resources and Life Sciences Vienna Austria

**Keywords:** *Arabidopsis thaliana*, endoplasmic reticulum, ERAD, glycosylation, *Nicotiana benthamiana*, protein degradation, protein homeostasis

## Abstract

The endoplasmic reticulum (ER) utilizes ER‐associated degradation (ERAD), a highly conserved eukaryotic pathway, to eliminate misfolded or unassembled proteins and maintain protein homeostasis in cells. The clearance of misfolded glycoproteins involves several distinct steps, including the recognition of a specific glycan signal, retrotranslocation to the cytosol, and subsequent degradation of the misfolded protein by the ubiquitin proteasome system. Confocal microscopy was used to track the fate of a well‐characterized ERAD substrate via a self‐complementing split fluorescent protein assay. The results demonstrate that a misfolded variant of the STRUBBELIG (SUB) extracellular protein domain (SUBEX‐C57Y) is retrotranslocated to the cytosol when transiently expressed in *Nicotiana benthamiana* leaf epidermal cells. Retrotranslocation requires a protein domain with a lesion that is exposed in the lumen of the ER, N‐glycan trimming by α‐mannosidases, HRD1‐mediated ubiquitination, and the ATPase function of CDC48. The retrotranslocated SUBEX‐C57Y ERAD substrate undergoes deglycosylation, and proteasomal degradation is blocked by a catalytically inactive cytosolic peptide N‐glycanase. These findings define distinct aspects of ERAD that have been elusive until now and may represent the default pathway for degrading misfolded glycoproteins in plants.

## Significance Statement

ER‐associated degradation (ERAD) represents an important cellular pathway that ensures protein degradation and restores protein homeostasis. In this study, we employed a retrotranslocation assay in conjunction with a biochemical analysis to elucidate the role of distinct ER and cytosolic proteins during ERAD.

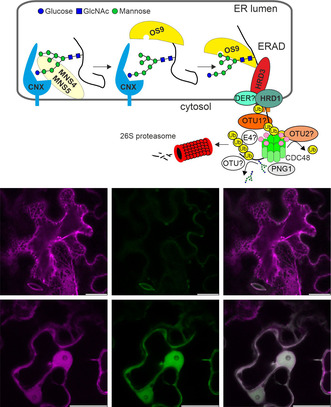

## INTRODUCTION

The endoplasmic reticulum (ER) is a major hub for protein biogenesis, post‐translational modifications, and trafficking of proteins to other organelles of the endomembrane system, the plasma membrane or the extracellular space. Protein folding is error prone and under various environmental stresses incompletely folded or terminally misfolded proteins can accumulate, threatening protein homeostasis of the ER and other organelles (Strasser, 2018). In eukaryotes, ER‐associated degradation (ERAD) is an important pathway for removal of aberrant or excess proteins from the ER to alleviate proteotoxic stress (Brandizzi, 2021; Christianson et al., 2023). Disposal of non‐membrane anchored luminal proteins requires either transport to other cellular organelles such as the vacuole or passage through the ER membrane to the cytosol, as the ER does not have its own protein degradation machinery. Fundamental steps of ERAD include the recognition of a lesion or degradation signal, targeting of the aberrant protein to a membrane‐embedded E3 ubiquitin ligase containing complex, retrotranslocation to the cytosol, polyubiquitination and delivery to the 26S proteasome (Smith et al., 2011; Vembar & Brodsky, 2008).

Our knowledge of the retrotranslocation of various glycan‐dependent ERAD substrates and the involvement of the proteasome in ERAD is still limited in plants (Guo et al., 2021; Hong et al., 2008, 2012; Hüttner, Veit, Vavra, Schoberer, Liebminger, et al., 2014; Niemann et al., 2018). Many ER quality control and ERAD factors were initially isolated based on *Arabidopsis thaliana bri1‐5* and *bri1‐9* suppressor screens (Hong et al., 2008, 2012; Jin et al., 2007; Su et al., 2011). In suppressor mutants, that are deficient in ERAD factors like the lectin OS9, the adaptor protein HRD3 (SEL1L in mammals), the E3 ubiquitin ligases HRD1A/HRD1B, or the α‐mannosidases MNS4/MNS5, partially misfolded but still functional BRASSINOSTEROID‐INSENSITIVE1 (BRI1) receptors BRI1‐5/BRI1‐9 accumulate in the ER. As a consequence of this accumulation, some of the aberrant BRI1 receptor can escape to the plasma membrane where they are functional and restore plant growth. Although many ER‐resident and ER membrane‐embedded proteins have been identified, no factors required for the retrotranslocation process or cytoplasmic proteins that block degradation and cause the accumulation of BRI1‐5/BRI1‐9 in the ER have been identified.

Much of the current understanding of ERAD is derived from studies focusing on the degradation of misfolded glycoproteins that are decorated with at least one N‐glycan (Hosokawa et al., 2009; Hüttner, Veit, Vavra, Schoberer, Dicker, et al., 2014; Spear & Ng, 2005). Selective trimming of a specific mannose residue by class I α‐mannosidases (MNS4 and MNS5 in Arabidopsis) (Hüttner, Veit, Vavra, Schoberer, Liebminger, et al., 2014) generates a glycan degradation signal on the ERAD substrate that is recognized by the ER lectin OS9 (Clerc et al., 2009; Quan et al., 2008). Interaction of the lectin‐misfolded protein complex with the membrane‐bound adaptor protein HRD3/SEL1L targets the ERAD substrate to the HRD1 E3 ubiquitin ligase ERAD complex. All these steps are highly conserved in eukaryotes and, except for initial recognition, well characterized in plants (Chen et al., 2020; Duan et al., 2023; Strasser, 2018).

Retrotranslocation and ubiquitination of misfolded soluble proteins are hallmarks of ERAD and are required to target proteins for proteasomal degradation in the cytosol. Despite the extensive knowledge of these late ERAD steps in yeast and mammalian cells (Li et al., 2024; Lopata et al., 2020; Preston & Brodsky, 2017; Rao et al., 2023; Wu & Rapoport, 2018), comparatively little is known in plants. Retrotranslocation has been demonstrated for overexpressed Arabidopsis CYTOKININ OXIDASE/DEHYDROGENASE1 (CKX1) (Guo et al., 2021), for a secreted form of GFP fused to the P domain of the ER‐resident lectin chaperone calreticulin (Brandizzi et al., 2003) and for ricin chains expressed in tobacco protoplasts (Di Cola et al., 2001, 2005; Marshall et al., 2008). Both ubiquitination and proteasome‐dependent degradation have been demonstrated for BRI1‐9 (Liu et al., 2015). In contrast, no proteasome‐dependent degradation has been shown so far for the ERAD substrates BRI1‐5 (Hong et al., 2008), and the soluble misfolded extracellular domain of Arabidopsis STRUBBELIG, termed SUBEX‐C57Y (Hüttner, Veit, Vavra, Schoberer, Dicker, et al., 2014). ER‐phagy or ER‐to‐lysosome/vacuole trafficking may serve as alternative disposal routes for misfolded glycoproteins that are not degraded by the proteasome (Rudinskiy & Molinari, 2023; Shin, Vavra, & Strasser, 2021), but this remains to be shown for potential ERAD substrates.

The data that are currently available are not consistent and do not provide sufficient information on the late stages of ERAD. In order to gain a better understanding of the degradation pathway of a misfolded soluble ERAD substrate, we have investigated previously poorly understood processes for the degradation of SUBEX‐C57Y. We established a retrotranslocation assay, investigated the ubiquitination of the ERAD substrate, and revealed the critical role of a cytosolic peptide N‐glycanase (PNGase). The data show that a misfolded soluble glycoprotein ERAD substrate is retrotranslocated in plants, confirm its ubiquitination and provide evidence for deglycosylation prior to proteasomal degradation.

## RESULTS

### SUBEX‐C57Y is retrotranslocated to the cytosol

**Figure 1:**
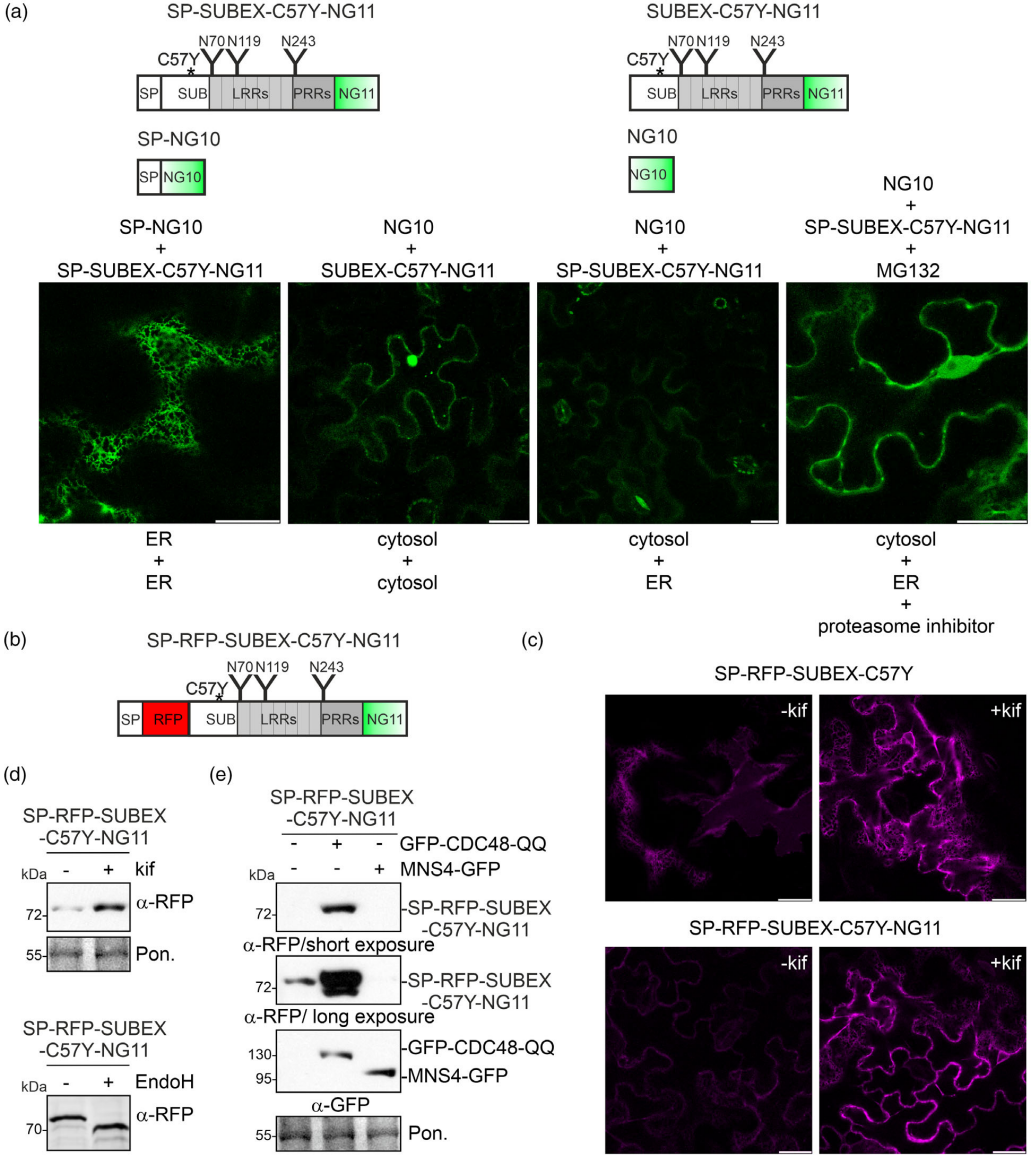
A split‐mNeonGreen2 reconstitution assay reveals the retrotranslocation of ER‐targeted SUBEX‐C57Y. (a) Schematic representation of the expressed SUBEX‐C57Y proteins. The C57Y mutation in the extracellular domain of STRUBBELIG (SUBEX) is indicated by an asterisk. N‐glycosylation sites are represented by “Y” and their amino acid positions are depicted. SP, STRUBBELIG signal peptide; SUB, STRUBBELIG‐domain; LRRs, leucine‐rich repeats; PRRs, proline‐rich repeats; NG11, mNeonGreen2 β‐sheet part 11; NG10, parts 1–10 of mNeonGreen2. The proteins were transiently expressed in *Nicotiana benthamiana* leaf epidermal cells and confocal microscopy images were taken 48 h after infiltration. MG132 (40 μm) was infiltrated 19 h prior to confocal microscopy. Scale bars = 10 μm. (b) Schematic illustration of the SP‐RFP‐SUBEX‐C57Y‐NG11 protein. (c) Confocal images of ER‐targeted SP‐RFP‐SUBEX‐C57Y and SP‐RFP‐SUBEX‐C57Y‐NG11 in the absence/presence of 50 μM kifunensine (kif). Scale bars = 10 μm. (d) Immunoblot analysis of SP‐RFP‐SUBEX‐C57Y‐NG11 shown in (c). Immunoblot of EndoH digested SP‐RFP‐SUBEX‐C57Y‐NG11. (e) The indicated proteins were transiently co‐expressed in *N. benthamiana* leaves and protein extracts were subjected to reducing SDS‐PAGE before immunoblotting with the indicated antibodies. Pon, Ponceau S staining of the membrane.

A self‐complementing split fluorescent protein (Zhong & Fang, 2012) was used to investigate the late steps of the ERAD pathway in plants (Duan et al., 2023; Vembar & Brodsky, 2008), and image retrotranslocation in *Nicotiana benthamiana* leaf epidermal cells. The misfolded SUBEX‐C57Y protein was fused to the 16 amino acid long sequence forming the 11th β sheet of mNeonGreen2 (NG11) (Figure 1a) (Feng et al., 2017; Stoddard & Rolland, 2019) and the fusion protein SUBEX‐C57Y‐NG11 was either targeted to the secretory pathway by the attachment of the N‐terminal STRUBBELIG signal peptide (SP‐SUBEX‐C57Y‐NG11) or expressed without any signal peptide (SUBEX‐C57Y‐NG11) to achieve cytosolic expression. The complementary parts 1–10 of mNeonGreen2 were either expressed in the cytosol (NG10) or targeted to the secretory pathway by fusion to the signal peptide of barley α‐amylase (SP‐NG10). Transient expression of both ER‐targeted proteins (SP‐NG10 co‐expressed with SP‐SUBEX‐C57Y‐NG11) in *N. benthamiana* leaf epidermal cells revealed specific subcellular localization in a network‐like structure as typical for the ER (Figure 1a). Co‐expression of the cytosol‐targeted NG10 with SUBEX‐C57Y‐NG11 lacking a signal peptide resulted in cytosolic localization and additional fluorescence was detected in the nucleus. When NG10 and SP‐SUBEX‐C57Y‐NG11 were co‐expressed, hardly any fluorescence signal was obtained. However, co‐expression of the same proteins in the presence of the proteasome inhibitor MG132 resulted in cytosolic and nuclear labelling, indicating self‐complementation of the fluorescent protein. These data suggest that SP‐SUBEX‐C57Y‐NG11 is translocated from the ER to the cytosol and subjected to proteasomal degradation.

To monitor the subcellular localization of the misfolded protein during ERAD at different stages, an additional construct that expresses a variant of SP‐SUBEX‐C57Y‐NG11 with RFP inserted between the signal peptide and the SUBEX‐C57Y sequence was created (Figure 1b). The detection of RFP fluorescence indicated that SP‐RFP‐SUBEX‐C57Y‐NG11 is located in the ER when transiently expressed in *N. benthamiana* leaf epidermal cells (Figure 1c). Additionally, both imaging and immunoblotting of total soluble proteins extracted from infiltrated leaves demonstrated that SP‐RFP‐SUBEX‐C57Y‐NG11 was stabilized by kifunensine, a known inhibitor of glycan‐dependent ERAD (Hüttner, Veit, Vavra, Schoberer, Liebminger, et al., 2014) and behaved similarly to the previously characterized ERAD substrate SP‐RFP‐SUBEX‐C57Y lacking the NG11 domain (Shin et al., 2018) (Figure 1c,d). SP‐RFP‐SUBEX‐C57Y‐NG11 is glycosylated with oligomannosidic N‐glycans, which is typical for ER‐retained proteins. This was demonstrated through endoglycosidase H (EndoH)‐mediated removal of N‐glycans (Figure 1c). Expression of a dominant variant of the ATPase CDC48A (CDC48‐QQ mutant, deficient in ATPase activity) which blocks ERAD (Marshall et al., 2008; Müller et al., 2005), resulted in accumulation of SP‐RFP‐SUBEX‐C57Y‐NG11, whereas increased clearance was observed by co‐expression of the α‐mannosidase MNS4, which promotes ERAD of misfolded glycoproteins by generating the glycan degradation signal (Hüttner, Veit, Vavra, Schoberer, Liebminger, et al., 2014) (Figure 1e). The additional faster migrating SP‐RFP‐SUBEX‐C57Y‐NG11 band, which was identified following prolonged exposure, does not correspond to a deglycosylated variant and remains susceptible to Endo H digestion (Figure S1). Taken together, these results show that SP‐RFP‐SUBEX‐C57Y‐NG11, like SP‐SUBEX‐C57Y‐GFP (Hüttner, Veit, Vavra, Schoberer, Dicker, et al., 2014) and SP‐RFP‐SUBEX‐C57Y (Shin et al., 2018), is a glycan‐dependent ERAD substrate.

**Figure 2:**
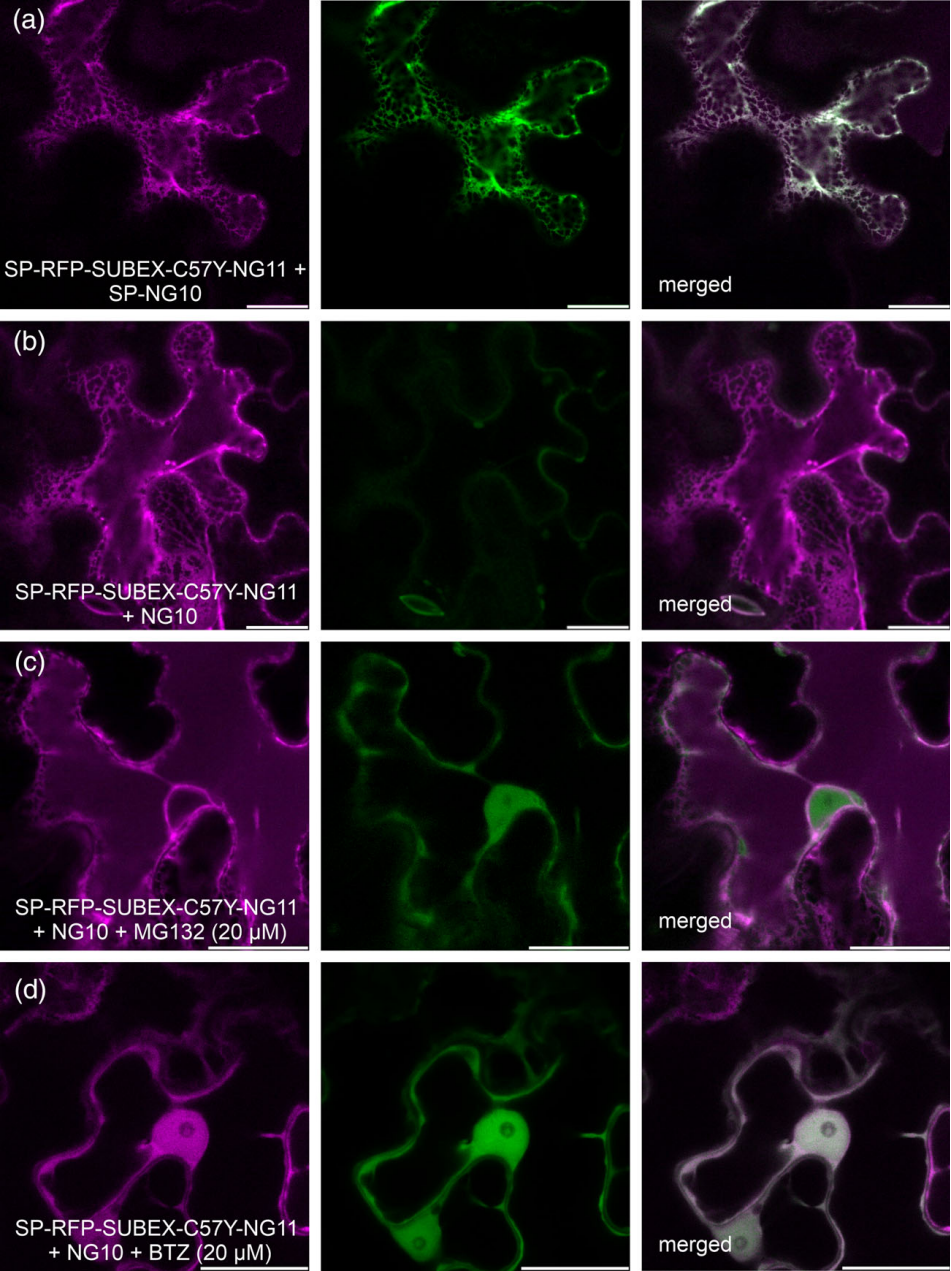
Specific proteasome inhibition results in accumulation of SUBEX‐C57Y in the cytosol and nucleus. (a) Representative confocal images of SP‐RFP‐SUBEX‐C57Y‐NG11 transiently co‐expressed with SP‐NG10 in *Nicotiana benthamiana* leaf epidermal cells. (b) SP‐RFP‐SUBEX‐C57Y‐NG11 co‐expressed with NG10. (c) SP‐RFP‐SUBEX‐C57Y‐NG11 co‐expressed with NG10 and 20 μm MG132. (d) SP‐RFP‐SUBEX‐C57Y‐NG11 co‐expressed with NG10 and 20 μm bortezomib (BTZ). Scale bars = 10 μm.

When ER‐targeted SP‐RFP‐SUBEX‐C57Y‐NG11 was co‐expressed with ER‐targeted SP‐NG10, both fluorescence signals were detected in the ER (Figure 2a). Upon co‐expression of SP‐RFP‐SUBEX‐C57Y‐NG11 and the cytosolic NG10, SP‐RFP‐SUBEX‐C57Y‐NG11 was mainly found in the ER, but no specific green fluorescence signal was detected in the cytosol, nucleus, or any other organelle (Figure 2b). However, when the proteasome inhibitor MG132 was present, green fluorescence was observed in the cytosol and nucleus when SP‐RFP‐SUBEX‐C57Y‐NG11 was co‐expressed with cytosolic NG10 (Figure 2c). This provides additional evidence for retrotranslocation of the ERAD substrate. As a control, leaf epidermal cells were infiltrated with kifunensine followed by MG132 infiltration. Under these experimental conditions, only few cells with green fluorescence in the nucleus were found indicating that mannose trimming is a prerequisite for retrotranslocation (Figure S2). As the cellular response to proteasome inhibitors can vary, cytosolic NG10 and SP‐RFP‐SUBEX‐C57Y‐NG11 was co‐expressed with the proteasome inhibitor bortezomib (BTZ). In the presence of this specific proteasome inhibitor, both red and green fluorescence were observed in the cytosol and nucleus (Figure 2d). The labelling of cytosol and nucleus with fluorescence in the presence of BTZ was prevented by inhibiting α‐mannosidases using kifunensine (Figure S2).

**Figure 3:**
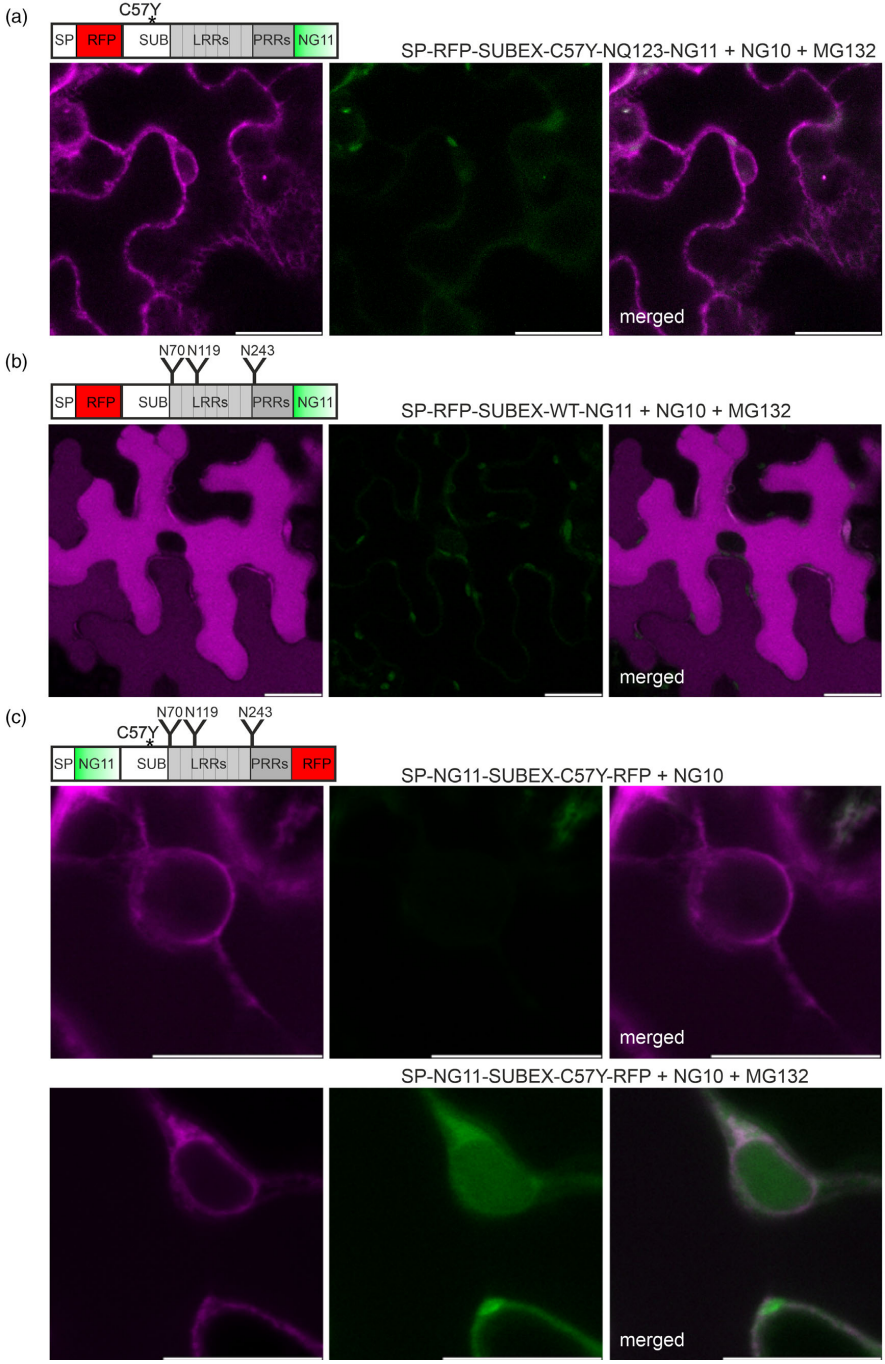
Efficient retrotranslocation of SUBEX‐C57Y is dependent on N‐glycosylation and the presence of a misfolded protein domain. (a) Representative confocal images of SP‐RFP‐SUBEX‐C57Y‐NQ123‐NG11 transiently co‐expressed in *Nicotiana benthamiana* leaf epidermal cells with NG10 and 40 μm MG132. (b) SP‐RFP‐SUBEX‐WT‐NG11 co‐expressed with NG10 and 40 μm MG132. (c) SP‐NG11‐SUBEX‐C57Y‐RFP co‐expressed with NG10 in the presence or absence of 40 μm MG132. The protein domain architecture is indicated in the schematic illustrations. Scale bars = 10 μm.

To investigate the dependence on glycosylation and misfolding, the retrotranslocation experiment was performed with the misfolded non‐glycosylated SP‐RFP‐SUBEX‐C57Y‐NQ123‐NG11 and the non‐mutated glycosylated SP‐RFP‐SUBEX‐WT‐NG11 protein (Hüttner, Veit, Vavra, Schoberer, Dicker, et al., 2014). While the misfolded non‐glycosylated variant was mainly found in the ER (Figure 3a; Figure S3), virtually no green fluorescence was observed in the cytosol and nucleus when NG10 was co‐expressed with MG132. SP‐RFP‐SUBEX‐WT‐NG11 was mainly found in the vacuole. As with SP‐RFP‐SUBEX‐C57Y‐NQ123‐NG11, no fluorescence signal was detected in the cytosol or nucleus in the presence of MG132 (Figure 3b). However, co‐expression of SP‐NG10 resulted in green fluorescence detection in the ER, indicating that mNeonGreen2 reconstitution is possible with both the misfolded non‐glycosylated and the non‐mutated glycosylated SUBEX variants when the fusion proteins carrying fluorescent protein domains are expressed in the same subcellular compartment (Figure S3). The results suggest that the retrotranslocation of the SUBEX‐C57Y ERAD substrate depends on N‐glycosylation and the recognition of a structurally defective protein in the ER.

The split fluorescence self‐complementation assay clearly showed that the C‐terminal part of the ERAD substrate carrying the NG11 peptide is retrotranslocated to the cytoplasm. To investigate whether the N‐terminal part of the fusion protein also undergoes retrotranslocation, the NG11 peptide was placed between the signal peptide and the misfolded SUBEX‐C57Y domain and RFP was attached to the C terminus. When expressed alone, SP‐NG11‐SUBEX‐C57Y‐RFP was mainly found in the ER (Figure S4). In the presence of MG132, co‐expression of cytosolic NG10 and SP‐NG11‐SUBEX‐C57Y‐RFP resulted in the formation of a green fluorescence signal in the nucleus (Figure 3c), as previously observed for SP‐RFP‐SUBEX‐C57Y‐NG11. This finding is consistent with the detection of red fluorescence derived from SP‐RFP‐SUBEX‐C57Y‐NG11 in the cytosol and nucleus in the presence of BTZ (Figure 2d), and demonstrates that both the N‐ and C terminus of a misfolded soluble ERAD substrate are translocated to the cytoplasm in plants.

### SUBEX‐C57Y degradation involves the HRD1 E3 ubiquitin ligase complex and the proteasome

**Figure 4:**
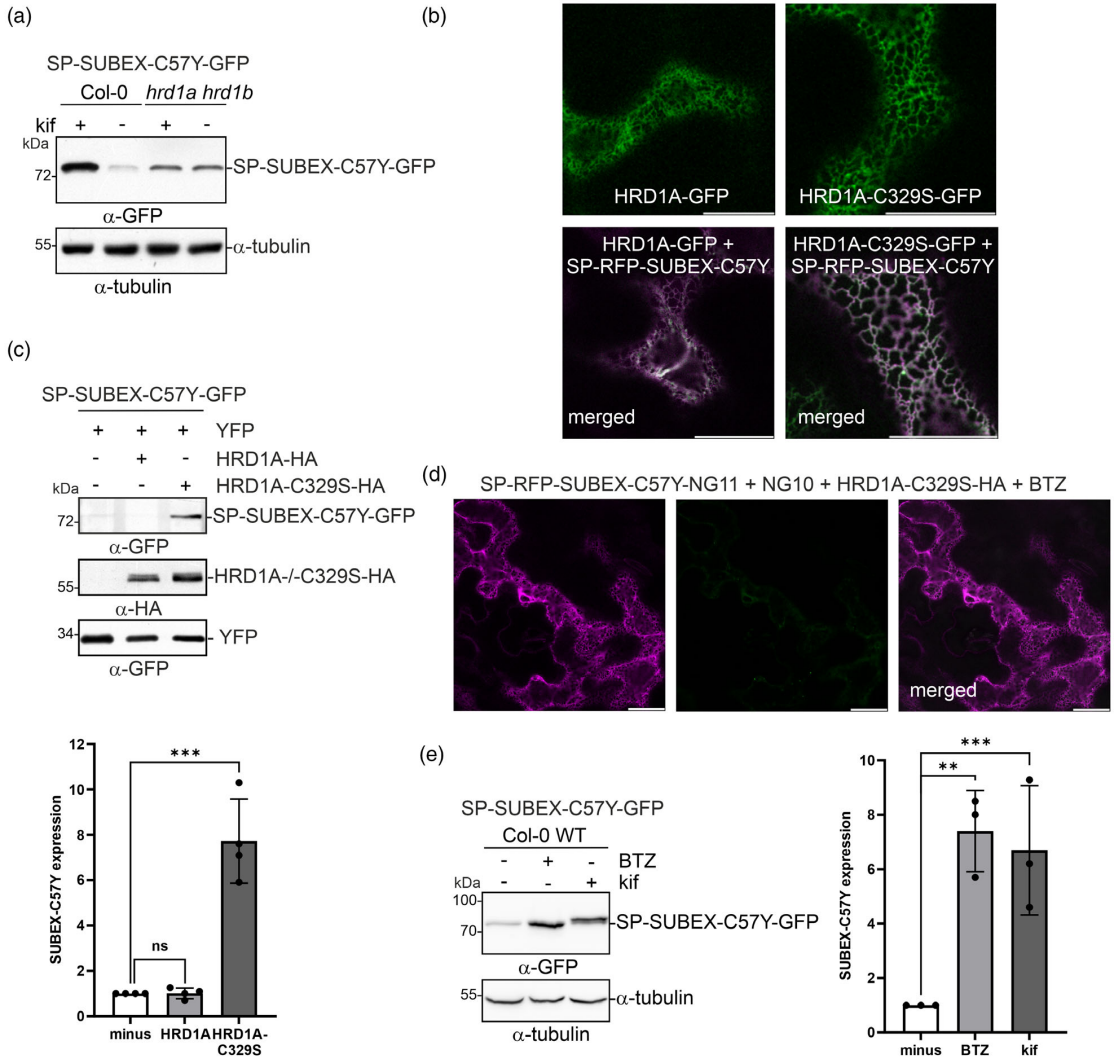
SUBEX‐C57Y degradation is mediated by the HRD1 E3 ubiquitin ligase. (a) Immunoblot analysis of SP‐SUBEX‐C57Y‐GFP expression in *Arabidopsis thaliana* Col‐0 WT or *hrd1a hrd1b* double mutant. Seedlings were incubated with or without 50 μm kifunensine (kif) for 24 h and subjected to immunoblotting with GFP and α‐tubulin antibodies. (b) Representative confocal images of HRD1A‐GFP and HRD1A‐C329S‐GFP transiently expressed in *Nicotiana benthamiana* leaf epidermal cells. In the merged image, HRD1A‐ or HRD1A‐C329S‐GFP was co‐expressed with SP‐RFP‐SUBEX‐C57Y. Scale bars = 10 μm. (c) Immunoblot analysis of SP‐SUBEX‐C57Y‐GFP co‐expressed in *N. benthamiana* leaves with HRD1A‐HA or HRD1A‐C329S‐HA. For quantification, the SP‐SUBEX‐C57Y‐GFP signals on the immunoblot were normalized to the co‐expressed YFP. Bars represent mean values ± SD (*n* = 4), significance levels are shown according to a Student's *t* test; ns, not significant, ***P < 0.001. (d) Representative confocal images of SP‐RFP‐SUBEX‐C57Y‐NG11 co‐expressed in *N. benthamiana* leaf epidermal cells with NG10, HRD1A‐C329S‐HA, and bortezomib (BTZ, 20 μm). Scale bars = 10 μm. (e) Immunoblot analysis of SP‐SUBEX‐C57Y‐GFP expression in *A. thaliana* Col‐0 WT after incubation of seedlings for 24 h with 50 μm kif or 20 μm BTZ. Detection of endogenous α‐tubulin was used as a loading control. For quantification, the immunoblot signals of SP‐SUBEX‐C57Y‐GFP were normalized to endogenous α‐tubulin expression. Bars represent mean values ± SD (*n* = 3), significance levels are shown according to a Student's *t* test; **P < 0.01, ***P < 0.001.

In a previous study, it was reported that the degradation of SUBEX‐C57Y depends on the processing of the mannosidic N‐glycans by the α‐mannosidases MNS4 and MNS5, the lectin OS9, and the ERAD adaptor protein HRD3/SEL1L (Hüttner, Veit, Vavra, Schoberer, Dicker, et al., 2014). In yeast, mammals, and plants, these proteins act in concert with the E3 ubiquitin ligase HRD1 to form the major ERAD complex involved in the degradation of soluble misfolded proteins (Chen et al., 2016; Christianson et al., 2008; Gauss et al., 2006; Su et al., 2011). The interaction of Arabidopsis HRD3/SEL1L with the lectin OS9, as well as the association between rice HRD3/SEL1L and HRD1, has been demonstrated in previous studies (Hüttner et al., 2012; Ohta & Takaiwa, 2015). Consistent with this finding, Arabidopsis HRD3/SEL1L was found to interact with HRD1 (Figure S5). To confirm the involvement of HRD1 in ERAD of SUBEX‐C57Y, SP‐SUBEX‐C57Y‐GFP was expressed in the Arabidopsis *hrd1a hrd1b* double mutant. Consistent with previous findings for *mns4 mns5*, *os9*, and *hrd3/sel1l* (Hüttner, Veit, Vavra, Schoberer, Dicker, et al., 2014), SP‐SUBEX‐C57Y‐GFP was easily detectable *hrd1a hrd1b* and upon treatment with kifunensine, no additional accumulation of the misfolded protein was observed (Figure 4a). This suggests that the two functionally redundant Arabidopsis HRD1 E3 ubiquitin ligases HRD1A/HRD1B play a crucial role in ERAD of SP‐SUBEX‐C57Y‐GFP. Next, HA‐ and GFP‐tagged versions of HRD1A were created, as well as an E3‐defective enzyme (HRD1A‐C329S, a variant that is dominant negative and deficient in E3 ubiquitin ligase activity, resulting from a mutation of a crucial cysteine residue within the RING domain) (Kaneko et al., 2002). HRD1A‐GFP and HRD1A‐C329S‐GFP variants transiently expressed in *N. benthamiana* were found in the ER and both showed co‐localization with SP‐RFP‐SUBEX‐C57Y (Figure 4b). In line with the findings from the *hrd1a hrd1b* mutant, the co‐expression of HRD1A‐C329S‐HA blocked the degradation of SP‐SUBEX‐C57Y‐GFP and resulted in a significant accumulation of SP‐SUBEX‐C57Y‐GFP (Figure 4c). Furthermore, HRD1A‐C329S‐HA expression resulted in an increased fluorescence signal of SP‐NG11‐SUBEX‐C57Y‐RFP in the ER without any cytosolic or nuclear labelling in the presence (Figure 4d) or absence (Figure S6) of BTZ. Taken together, these results demonstrate that SUBEX‐C57Y variants are degraded by the HRD1 ERAD complex in plants.

In a previous study, it was found that there was no significant increase of SP‐SUBEX‐C57Y‐GFP when Arabidopsis wild type were incubated with MG132 (Hüttner, Veit, Vavra, Schoberer, Dicker, et al., 2014). The transient infiltration experiment with BTZ indicated that this specific inhibitor is more potent than MG132 in blocking of the proteasome. Treatment of Arabidopsis Col‐0 wild‐type seedlings with BTZ resulted in a significant increase in SP‐SUBEX‐C57Y‐GFP protein levels, similar to the increase observed with kifunensine treatment (Figure 4e). This indicates that SP‐SUBEX‐C57Y‐GFP is degraded by the proteasome.

### SUBEX‐C57Y is ubiquitinated

**Figure 5:**
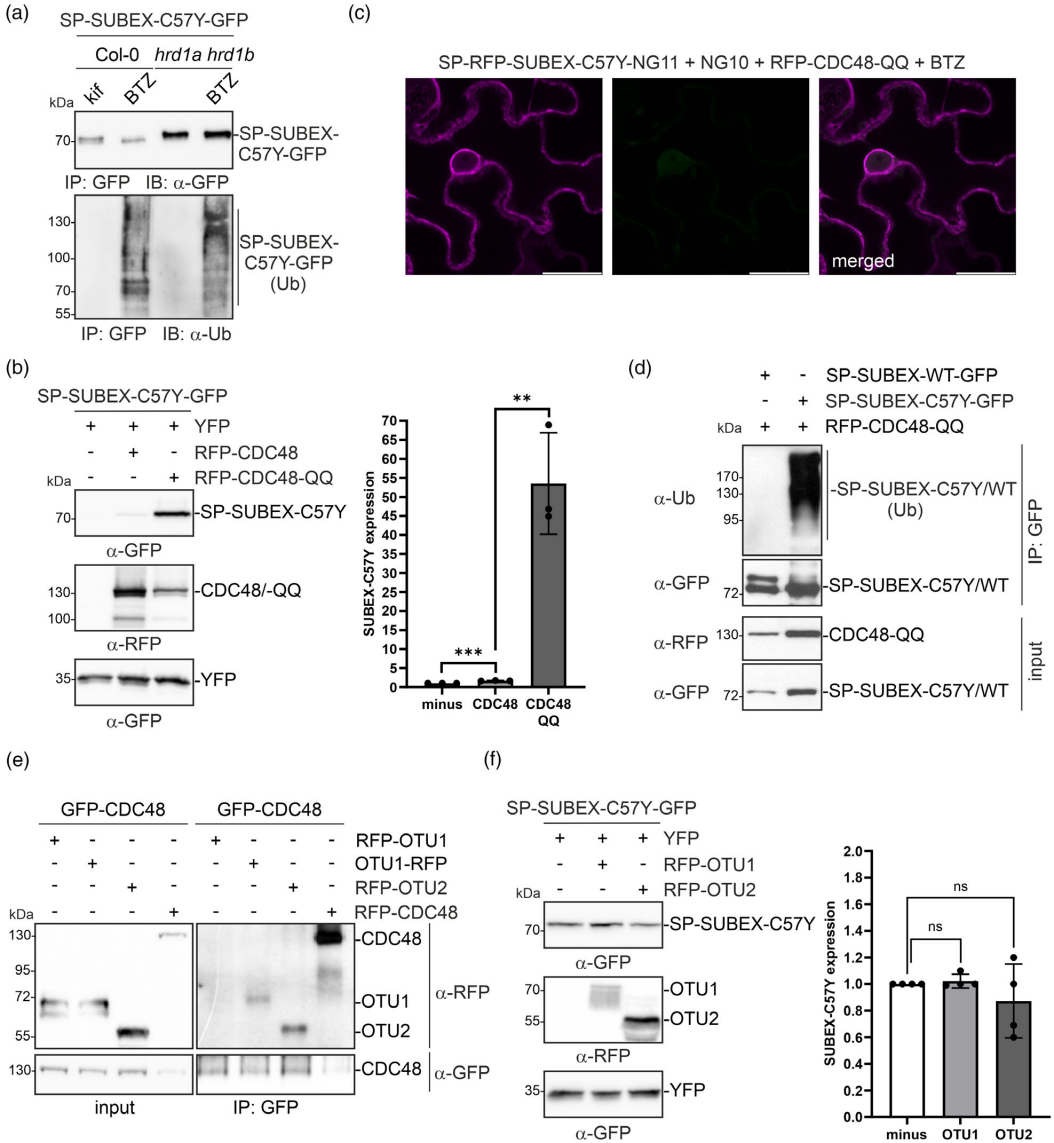
SUBEX‐C57Y is ubiquitinated. (a) Immunoblot analysis of SP‐SUBEX‐C57Y‐GFP purified from *Arabidopsis* Col‐0 WT seedlings incubated for 24 h with 50 μm kifunensine (kif) or 20 μm bortezomib (BTZ) or from the *hrd1a hrd1b* double mutant incubated without or with BTZ. Proteins were detected with GFP and ubiquitin‐specific (α‐Ub) antibodies. (b) Immunoblot analysis of SP‐SUBEX‐C57Y‐GFP transiently co‐expressed in *Nicotiana benthamiana* leaves with RFP‐CDC48 or with RFP‐CDC48‐QQ. Co‐expressed YFP was used for normalization of the SP‐SUBEX‐C57Y‐GFP expression. Bars represent mean values ± SD (*n* = 3), significance levels are shown according to a Student's *t* test; **P < 0.01, ***P < 0.001. (c) Representative confocal images of SP‐RFP‐SUBEX‐C57Y‐NG11 co‐expressed in *N. benthamiana* leaf epidermal cells with NG10 and RFP‐CDC48‐QQ in the presence of BTZ. Scale bars = 10 μm. (d) Immunoblot analysis of SP‐SUBEX‐WT‐GFP and SP‐SUBEX‐C57Y‐GFP transiently co‐expressed in *N. benthamiana* leaves with RFP‐CDC48‐QQ. SUBEX‐variants were purified using GFP‐trap beads and subjected to immunoblotting with GFP and ubiquitin‐specific (α‐Ub) antibodies. (e) Immunoblot analysis of GFP‐trap purified GFP‐CDC48 transiently co‐expressed with RFP‐OTU1, OTU1‐RFP, RFP‐OTU2 or RFP‐CDC48. (f) Immunoblot analysis of SP‐SUBEX‐C57Y‐GFP transiently co‐expressed with RFP‐OTU1 or with RFP‐OTU2. Co‐expressed YFP was used for normalization of the SP‐SUBEX‐C57Y‐GFP expression. Bars represent mean values ± SD (*n* = 4), significance levels are shown according to a Student's *t* test; ns, not significant.

The retrotranslocation assay and the inhibition of the proteasomal degradation provided evidence for clearance of the misfolded glycoprotein in the cytosol. In mammals, the extraction of ERAD substrates from the membrane by the cytosolic CDC48/p97/VCP ATPase complex and the subsequent proteasomal degradation requires polyubiquitination (Bodnar & Rapoport, 2017; Twomey et al., 2019). Therefore, it was investigated whether ubiquitination plays a role in the degradation of SUBEX‐C57Y variants. When SP‐SUBEX‐C57Y‐GFP was purified from Arabidopsis Col‐0 wild type treated with BTZ, ubiquitin was detected. This was not observed in seedlings incubated with kifunensine or SP‐SUBEX‐C57Y‐GFP purified from *hrd1a hrd1b* plants (Figure 5a). When *hrd1a hrd1b* were incubated with BTZ, ubiquitin was also detected upon purification of SP‐SUBEX‐C57Y‐GFP. However, in relation to the purified SP‐SUBEX‐C57Y‐GFP, the ubiquitin levels were reduced compared to Col‐0 treated with BTZ, and different bands were observed on the immunoblots. This suggests that another E3 ubiquitin ligase may modify the misfolded protein when the proteasome is inhibited.

Previous experiments have demonstrated that RFP‐CDC48‐QQ inhibits the degradation of ERAD substrates (Figure 1e). Additionally, co‐expression of RFP‐CDC48‐QQ resulted in a significant accumulation of SP‐SUBEX‐C57Y‐GFP (Figure 5b). However, co‐expression of SP‐RFP‐SUBEX‐C57Y‐NG11 in *N. benthamiana* epidermal cells with NG10 and RFP‐CDC48‐QQ did not lead to cytosolic or nuclear accumulation of green fluorescence, regardless of the presence (Figure 5c) or absence (Figure S7) of BTZ. This suggests that RFP‐CDC48‐QQ blocks the retrotranslocation.

To examine the impact of RFP‐CDC48‐QQ on ubiquitination, RFP‐CDC48‐QQ was co‐expressed with either SP‐SUBEX‐C57Y‐GFP or SP‐SUBEX‐WT‐GFP. The GFP‐tagged proteins were then purified and subjected to immunoblotting using a ubiquitin‐specific antibody (Figure 5d). Ubiquitination was only detected for the misfolded glycoprotein. Moreover, ubiquitinated SP‐RFP‐SUBEX‐C57Y was not detected when HRD1A‐C329S‐GFP was co‐expressed (Figure S8). This suggests that retrotranslocation and ubiquitination require the initial recognition of a misfolded protein in the ER, followed by mannose trimming and a functional HRD1 E3 ubiquitin ligase to mediate ubiquitination prior to extraction by CDC48.

After confirming the ubiquitination of SP‐SUBEX‐C57Y‐GFP, it was investigated whether deubiquitinating enzymes are involved in the degradation process. Ubiquitination may play a dual role, upstream and downstream of CDC48, as removal of ubiquitin interferes with recruitment of the CDC48 complex and/or the subsequent targeting to the cytosolic proteasome (Christianson et al., 2023; Ernst et al., 2009, 2011). The impact of Arabidopsis ovarian tumor family deubiquitinases OTU1 (*At1g28120*) (Zang et al., 2020) and OTU2 (*At1g50670*) (Radjacommare et al., 2014) on the degradation of SUBEX‐C57Y was investigated. OTU2 is a homolog of human YOD1 which is associated with the mammalian CDC48/p97/VCP ATPase complex. A dominant negative YOD1 variant blocked the degradation of misfolded glycoproteins in mammalian cells (Ernst et al., 2009). OTU1, on the other hand, is involved in the degradation of misfolded non‐glycosylated MLO variants in plants (Zang et al., 2020). To determine whether OTU1 and/or OTU2 are associated with CDC48, they were transiently co‐expressed with CDC48. CDC48 was purified and examined for co‐purification of OTU1 and OTU2 variants. Both deubiquitinases were found in the cytosol (Figure S9) and interacted with CDC48 (Figure 5e). Co‐expression of RFP‐OTU1 or RFP‐OTU2 did not affect the levels of SP‐SUBEX‐C57Y‐GFP protein (Figure 5f) or lead to the accumulation of SP‐RFP‐SUBEX‐C57Y‐NG11 in the cytosol or nucleus (Figure S9).

**Figure 6:**
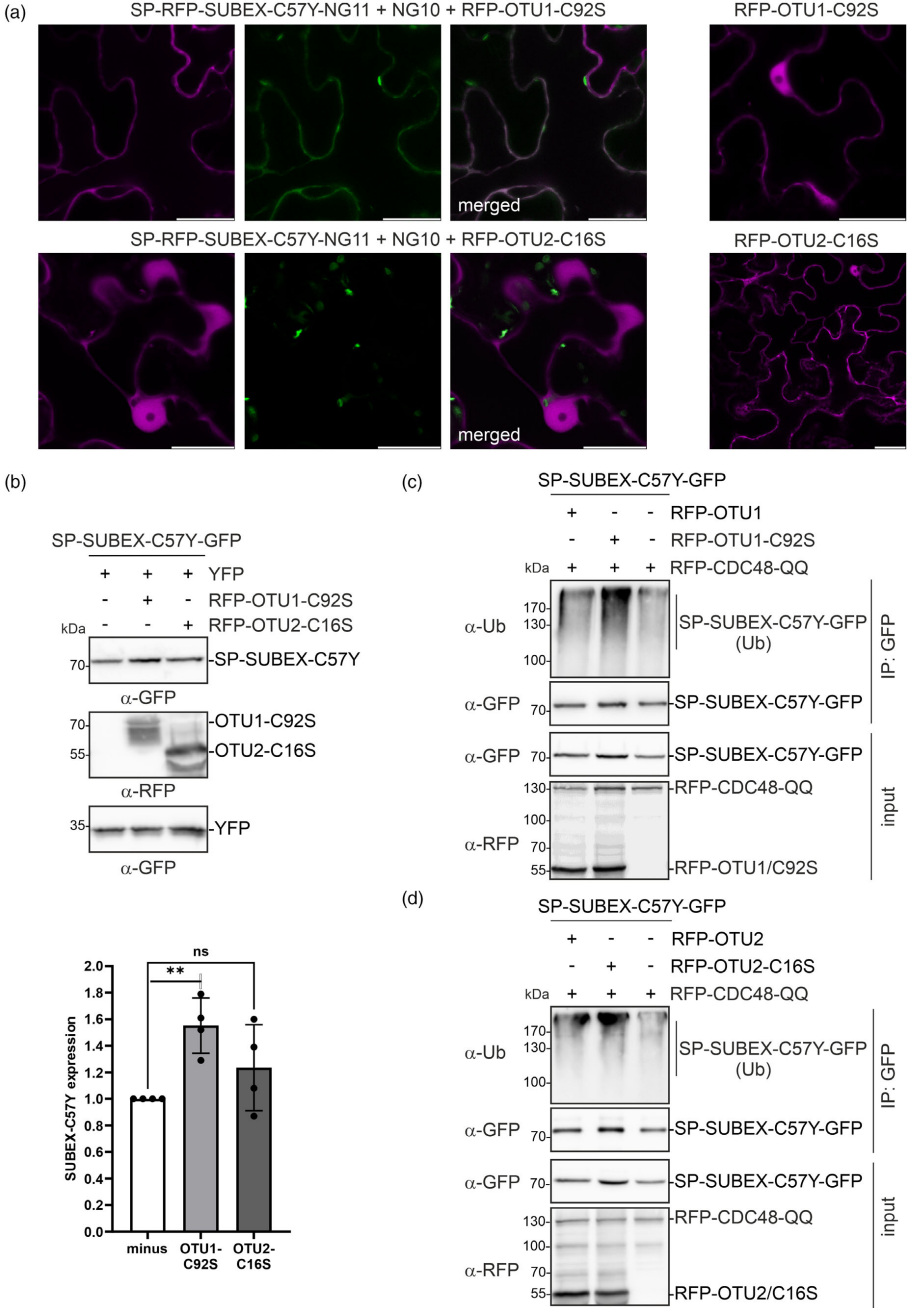
The expression of plant OTU1/OTU2 deubiquitinases plays a minor role in the degradation of misfolded SUBEX‐C57Y and its ubiquitination. (a) Representative confocal images of SP‐RFP‐SUBEX‐C57Y‐NG11 transiently co‐expressed in *Nicotiana benthamiana* leaf epidermal cells with NG10 and RFP‐OTU1‐C92S or RFP‐OTU2‐C16S. Scale bars = 10 μm. (b) Immunoblot analysis of SP‐SUBEX‐C57Y‐GFP transiently co‐expressed in *N. benthamiana* leaves with RFP‐OTU1‐C92S or RFP‐OTU2‐C16S. Co‐expressed YFP was used for normalization of the SP‐SUBEX‐C57Y‐GFP expression. Bars represent mean values ± SD (*n* = 4), significance levels are shown according to a Student's *t* test; ns, not significant, **P < 0.01. (c) Immunoblot analysis of SP‐SUBEX‐C57Y‐GFP transiently co‐expressed in *N. benthamiana* leaves with RFP‐CDC48‐QQ and RFP‐OTU1 or RFP‐OTU1‐C92S. SP‐SUBEX‐C57Y‐GFP was purified using GFP‐trap beads and subjected to immunoblotting with GFP, RFP, and ubiquitin‐specific (α‐Ub) antibodies. (d) Immunoblot analysis of SP‐SUBEX‐C57Y‐GFP transiently co‐expressed in *N. benthamiana* leaves with RFP‐CDC48‐QQ and RFP‐OTU2 or RFP‐OTU2‐C16S. SP‐SUBEX‐C57Y‐GFP was purified using GFP‐trap beads and subjected to immunoblotting with GFP, RFP, and ubiquitin‐specific (α‐Ub) antibodies.

Next, catalytically defective variants of OTU1 (OTU1‐C92S, deubiquitinase‐deficient variant) (Zang et al., 2020) and OTU2 (OTU2‐C16S, deubiquitinase‐deficient variant) (Radjacommare et al., 2014) were examined for their effect on ERAD substrate retrotranslocation when expressed together with SP‐RFP‐SUBEX‐C57Y‐NG11 and NG10. The results showed that RFP‐OTU2‐C16S did not have any noticeable effect on the ERAD substrate (Figure 6a), the same result was obtained with another mutant OTU2 variant (RFP‐OTU2‐C63S, deficient in ubiquitin chain association) (Radjacommare et al., 2014) (Figure S10). By contrast, the expression of RFP‐OTU1‐C92S led to a slight accumulation of green fluorescence in the cytosol without any labelling of the nucleus (Figure 6a). In line with this discovery, RFP‐OTU1‐C92S resulted in a slight but significant accumulation of SP‐SUBEX‐C57Y‐GFP (Figure 6b). To investigate whether the overexpression of deubiquitinases results in a reduction in ubiquitin levels, SP‐SUBEX‐C57Y‐GFP was expressed with RFP‐CDC48‐QQ and either OTU1 or OTU2 variants. Co‐immunoprecipitation followed by immunoblotting demonstrated that the overall ubiquitination levels remained unchanged when RFP‐OTU1 or RFP‐OTU2 variants were co‐expressed (Figure 6c,d; Figure S11). The co‐expression of RFP‐OTU1‐C92S resulted in the detection of slightly elevated levels of ubiquitinated protein. This increase appeared to be correlated with the slightly higher amounts of purified SP‐SUBEX‐C57Y‐GFP. Taken together, these results suggest that both deubiquitinases may only remove ubiquitin from the ERAD substrate in specific linkages or may be involved in other deubiquitination processes.

### Retrotranslocated SUBEX‐C57Y is deglycosylated

**Figure 7:**
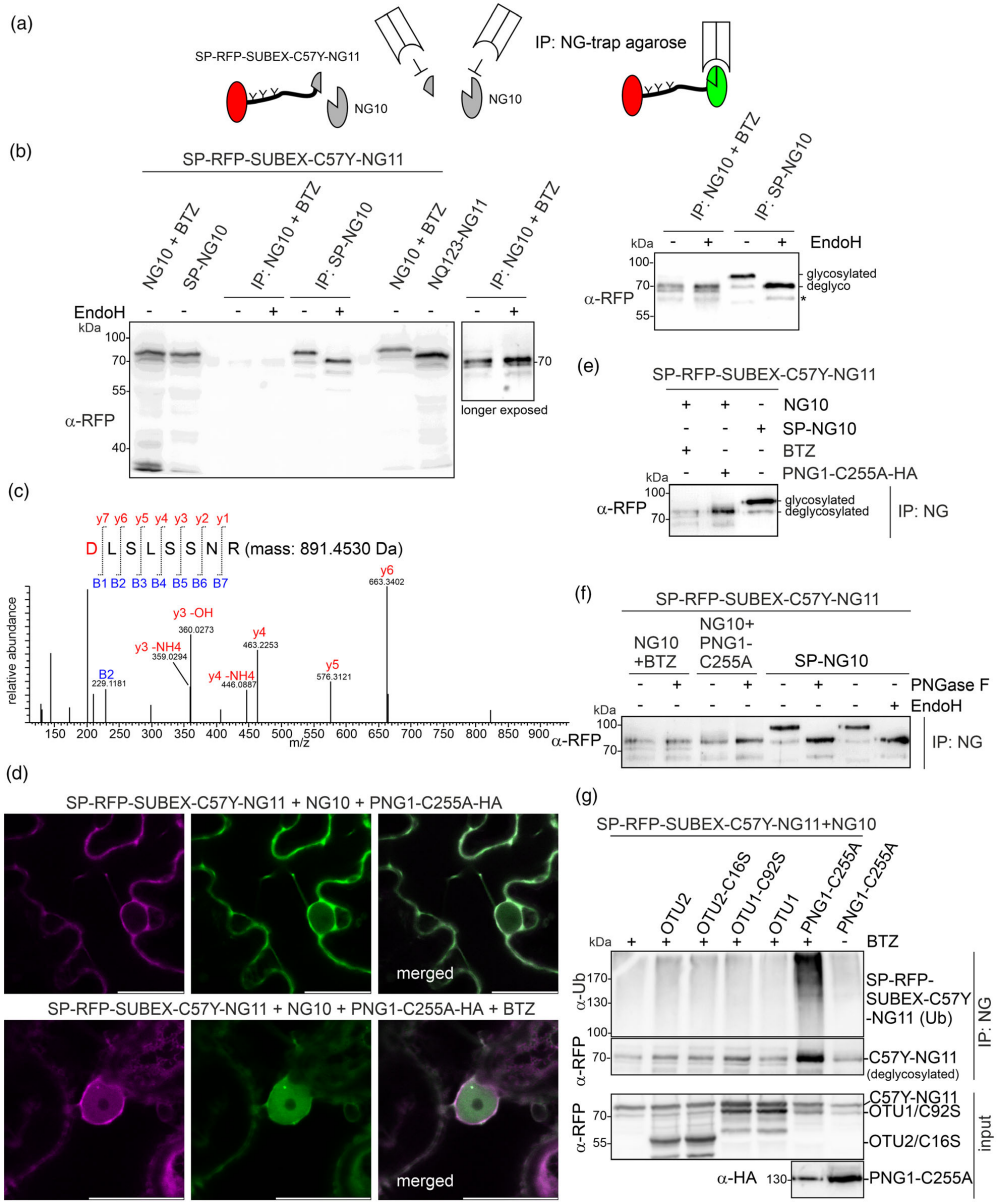
Retrotranslocated SUBEX‐C57Y is deglycosylated. (a) Schematic illustration of the capture approach used to purify reconstituted mNeonGreen2 (NG) fusion proteins. (b) Immunoblot analysis of NG‐trap (IP) purified SP‐RFP‐SUBEX‐C57Y‐NG11 transiently co‐expressed in *Nicotiana benthamiana* leaves with NG10 and BTZ (20 μm) or with SP‐NG10. Purified proteins were subjected to EndoH digestion to release the oligomannosidic N‐glycans. For comparison, protein extracts from the same samples prior to purification and the mobility of expressed SP‐RFP‐SUBEX‐C57Y‐NQ123‐NG11 are shown. The glycosylated and deglycosylated bands are marked. SP‐RFP‐SUBEX‐C57Y‐NG11 underwent deglycosylation either in *in vivo* during ERAD or *in vitro* upon EndoH digestion. (c) LC–MS/MS analysis of the deglycosylated SUBEX‐C57Y peptide carrying N‐glycosylation site 2 (due to the deglycosylation the peptide **N**LSLSSNR is converted to **D**LSLSSNR). Masses for diagnostic fragment ions are labeled. (d) Representative confocal images of SP‐RFP‐SUBEX‐C57Y‐NG11 transiently co‐expressed in *N. benthamiana* leaf epidermal cells with NG10 and PNG1‐C255A‐HA in the absence or presence of BTZ (20 μm). Scale bars = 10 μm. (e) Immunoblot analysis of NG‐trap purified SP‐RFP‐SUBEX‐C57Y‐NG11. SP‐RFP‐SUBEX‐C57Y‐NG11 was transiently co‐expressed in *N. benthamiana* leaves with either NG10 in the presence or absence of BTZ (20 μm) or with SP‐NG10. (f) Immunoblot analysis of endoglycosidase digestions of NG‐trap purified proteins. EndoH and PNGase F digestion of purified SP‐RFP‐SUBEX‐C57Y‐NG11 co‐expressed with SP‐NG10 is shown for comparison. (g) Immunoblot analysis of NG‐trap purified SP‐RFP‐SUBEX‐C57Y‐NG11 which was co‐expressed with NG10 and the indicated proteins (RFP‐OTU2, RFP‐OTU2‐C16S, RFP‐OTU1‐C92S, RFP‐OTU1, PNG1‐C255A‐HA).

SUBEX‐C57Y is a glycoprotein ERAD substrate with three N‐glycans (Hüttner, Veit, Vavra, Schoberer, Dicker, et al., 2014). Glycosylated proteins present an additional challenge to the degradation by the 26S proteasome and deglycosylation of glycoproteins by a cytosolic PNGase precedes proteasomal degradation (Hirsch et al., 2003). Consequently, deglycosylated intermediates may accumulate in the cytosol upon inhibition of proteolysis (Di Cola et al., 2005). To specifically isolate the cytosolic pool of SUBEX‐C57Y, SP‐RFP‐SUBEX‐C57Y‐NG11 was co‐expressed with NG10 in the presence of BTZ and the reconstituted NG fusion protein was purified (Figure 7a). Although the signal was weak on immunoblots detected with antibodies against RFP, the size of the purified protein was clearly different from the purified ER‐resident reconstituted protein (SP‐RFP‐SUBEX‐C57Y‐NG11 co‐expressed with SP‐NG10) and was comparable to SP‐RFP‐SUBEX‐C57Y‐NQ123‐NG11 which lacks N‐glycans (Figure 7b). Furthermore, digestion with EndoH did not result in any mobility shift indicating that the purified cytosolic fraction of SP‐RFP‐SUBEX‐C57Y‐NG11 is not glycosylated. Deglycosylation by PNGases converts asparagine to aspartate by deamidation (Hirayama et al., 2015), providing evidence that the absence of N‐glycans is due to a deglycosylation process in cells rather than incomplete N‐glycosylation in the ER (Castilho et al., 2018; Huang & Suzuki, 2020) or a SUBEX‐C57Y fusion protein that is not ER targeted (Grotzke et al., 2013). Therefore, purified SP‐RFP‐SUBEX‐C57Y‐NG11 was digested with trypsin and peptides were subjected to MS analysis to monitor differences in the mass of the SUBEX‐C57Y peptides bearing N‐glycosylation sites. Two of the three peptides were found which displayed masses (peptide mass + 1 Da) corresponding to asparagine to aspartate conversions, confirming the protein N‐glycosylation, the retrotranslocation and subsequent deglycosylation in the cytosol (Figure 7c; Figure S12).

Arabidopsis has only one cytosolic PNGase, PNG1 (Diepold et al., 2007), which acts on denatured glycoproteins *in vitro* (Shirai et al., 2021). A catalytic mutant of the Arabidopsis PNG1 enzyme (PNG1‐C251A, deficient in PNGase activity) inhibits the degradation of a glycosylated ERAD substrate in yeast (Masahara‐Negishi et al., 2012). Here, the study aimed to determine whether a catalytically defective PNG1 inhibits ERAD of SUBEX‐C57Y. To achieve this, a mutant of the putative *Nicotiana tabacum* PNG1 ortholog was created (PNG1‐C255A) and expressed as RFP or HA tagged variants. In *N. benthamiana* leaf epidermal cells, PNG1‐C255A‐RFP was located in the cytosol as anticipated for a deglycosylating enzyme involved in the proteasome degradation pathway (Figure S13). In the presence of PNG1‐C255A‐HA, SP‐RFP‐SUBEX‐C57Y‐NG11 co‐expressed with NG10 labeled the cytosol also in the absence of BTZ (Figure 7d), indicating a block of SP‐RFP‐SUBEX‐C57Y‐NG11 degradation in the cytosol. This NG‐fluorescence signal in the cytosol was even more pronounced when SP‐RFP‐SUBEX‐C57Y‐NG11 was co‐expressed with NG10 and the RFP‐tagged variant PNG1‐C255A‐RFP (Figure S13). The co‐expression of non‐mutated PNG1 did not have any impact on SUBEX‐C57Y (Figure S14), suggesting a specific block in the degradation process due to the overexpression of the catalytically inactive mutant PNG1‐C255A. It is worth noting that the effects of the proteasome inhibitor and PNG1‐C255A were different. Inhibiting the proteasome resulted in NG fluorescence in the cytosol, nuclear envelope and nucleoplasm. The inhibition of degradation through the co‐expression of PNG1‐C255A resulted in the labelling of the cytosol and nuclear envelope, but only weakly labeled the nucleoplasm (Figure 7d; Figure S13). In contrast to the effect observed for the retrotranslocated SUBEX‐C57Y variant, co‐expression of PNG1‐C255A‐RFP did not alter the protein levels of the glycosylated SUBEX‐C57Y, indicating that the processes are decoupled (Figure S13).

The glycosylated form of SP‐RFP‐SUBEX‐C57Y‐NG11 carrying mannosidic N‐glycans was not detected after purifying the reconstituted protein in the presence of PNG1‐C255A‐HA (Figure S15). On the immunoblots, the mobility of SP‐RFP‐SUBEX‐C57Y‐NG11 co‐expressed with PNG1‐C255A‐HA was similar to that of the protein purified in the presence of BTZ (Figure 7e) and neither EndoH nor PNGase F digestion resulted in an additional shift in mobility (Figure 7f). Consistent with the confocal data, we detected higher levels of the retrotranslocated ERAD substrate on immunoblots when PNG1‐C255A‐HA was co‐expressed in the presence of BTZ (Figure 7g). The increase in deglycosylated SP‐RFP‐SUBEX‐C57Y‐NG11 resulted in considerably higher levels of ubiquitinated protein.

Finally, it was investigated whether the results for SUBEX‐C57Y could be applied to other misfolded soluble glycoproteins. For this purpose, the N‐terminal region of Arabidopsis BRI1‐5 (NBRI1‐5) (Shin et al., 2018) fused to SP‐RFP and NG11 (SP‐RFP‐NBRI1‐5‐NG11) was co‐expressed with cytosolic NG10. Similarly to SUBEX‐C57Y, SP‐RFP‐NBRI1‐5‐NG11 was retrotranslocated in a glycan‐dependent manner (Figure S16), which also required a functional HRD1 (Figure S17). SP‐RFP‐NBRI1‐5‐NG11 was then degraded by the proteasome. SP‐RFP‐NBRI1‐5‐NG11 was deglycosylated and PNG1‐C255A‐HA co‐expression stabilized the protein predominantly in the cytosol (Figure S18). This suggests that the described ERAD pathway is commonly used for the degradation of structurally defective soluble glycoproteins.

## DISCUSSION

**Figure 8:**
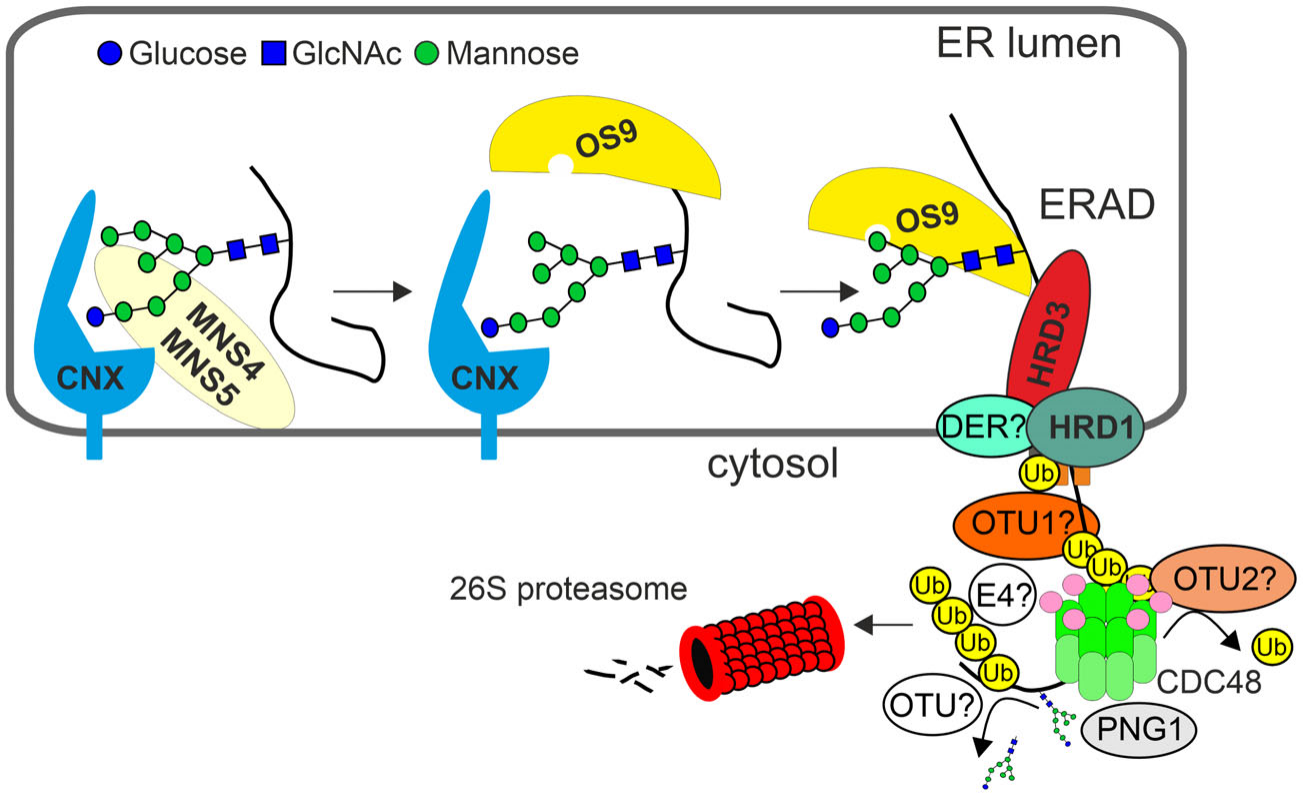
Current model for the degradation of luminal misfolded glycoproteins in plants. An ERAD substrate carrying at least one N‐glycan and an aberrant protein conformation that is recognized in the lumen of the ER is processed by the ER‐resident α‐mannosidase MNS4 or MNS5 that have overlapping functions. This results in the removal of the terminal α‐mannose residue from the C‐branch of the N‐glycan. The generated free α1,6‐linked mannose is then bound by the lectin OS9. OS9 interacts with the adaptor protein HRD3/SEL1L and targets the ERAD substrate to the membrane‐embedded HRD1 complex for retrotranslocation to the cytosol which is likely to involve other proteins such as DERLINs (DER). The substrate is subsequently ubiquitinated by the cytosolic E3 ubiquitin ligase domain of HRD1. OTU1 and/or OTU2 may contribute to removing specific ubiquitin monomers from the ERAD substrate or from a cytosolic ERAD component to fine‐tune protein degradation. The ERAD substrate, which has been polyubiquitinated, may be trimmed to oligoubiquitin chains by OTUs such as OTU1 or OTU2. The polypeptide chain is pulled out of the retrotranslocation channel by the CDC48 ATPase through a pore using a threading mechanism. The structurally defective polypeptide that exits at the *trans* side of the CDC48 complex (shown in light green) is likely to undergo ubiquitin extension or re‐modeling, which may involve one or more cytosolic E4 ubiquitin ligases that are yet unknown, as well as ubiquitin trimming by OTUs or other deubiquitinases. The cytosolic PNG1 removes the attached N‐glycans before the unfolded ERAD substrates enter the 26S proteasome.

In this study, we investigated the poorly understood processing steps involved in ERAD of soluble misfolded glycoproteins (Figure 8). Specifically, we investigated retrotranslocation, the role of HRD1 in ubiquitination, the function of CDC48, its relationship with two deubiquitinases, and the role of deglycosylation mediated by a cytosolic PNGase. After initial recognition of a soluble glycosylated ERAD client protein and mannose trimming by MNS4 or MNS5 in the lumen of the ER (Hüttner, Veit, Vavra, Schoberer, Liebminger, et al., 2014), the lectin OS9 binds to the generated α1,6‐mannose on the C branch of the N‐glycan and interacts with the adaptor protein HRD3/SEL1L to recruit clients to the HRD1 complex. In yeast, luminal ERAD substrates cross the membrane through a channel that is formed by HRD1 and the rhomboid‐like protein DER1 (Wu et al., 2020). The misfolded polypeptides are inserted into the channel as a loop. This finding is consistent with the observation that both the N‐ and C‐terminal ends of SUBEX‐C57Y are subsequently translocated to the cytosol. DER1 is also associated with ERAD in rice and interacts with the HRD1 complex (Qian et al., 2018), suggesting that the proposed retrotranslocation mechanism in yeast may be similar in plants. In this study, we did not specifically examine the retrotranslocation mechanism, but our split fluorescent protein reconstitution assay allowed us to monitor the fate of soluble ERAD substrates and their detection in the cytosol. In the future, the assay will enable the identification of currently unknown factors involved in the retrotranslocation process in plants.

Studies with yeast and metazoans suggest that distinct steps are required for the degradation of different glycoprotein ERAD substrates. For example, in *Drosophila* and mouse cells, deglycosylation of an ERAD substrate by the cytosolic PNGase was found to be associated with retrotranslocation with impaired deglycosylation at the ER membrane, leading to accumulation of misfolded proteins in the ER (Galeone et al., 2020). In another study, it was found that PNGase deficiency results in the detection of glycosylated proteins in the cytosol without any apparent effect on retrotranslocation and protein accumulation in the ER (Blom et al., 2004). Here, we observed that a block of ubiquitination by overexpression of a catalytically inactive HRD1 E3 ubiquitin ligase caused the accumulation of the glycosylated ERAD substrate in the ER. Overexpression of an enzymatically inactive CDC48 had a similar effect on ERAD client proteins and resulted in the accumulation of glycosylated SUBEX‐C57Y, suggesting that co‐expression of CDC48‐QQ results in a blockage of the dislocation process. However, SUBEX‐C57Y stabilized by the catalytically inactive CDC48‐QQ was ubiquitinated. This indicates that a segment of the misfolded polypeptide is exposed to the cytosol, where it is ubiquitinated by HRD1.

In contrast to the results observed for HRD1A‐C329A and CDC48‐QQ, overexpression of the mutant PNG1 variant did not result in a notable increase in the levels of the glycosylated SUBEX‐C57Y variant in the ER. The observations indicate that PNG1‐mediated deglycosylation occurs downstream of ubiquitination and is independent of the retrotranslocation process. This is in line with previous studies on the degradation of ricin toxin A in tobacco protoplasts (Di Cola et al., 2001, 2005). However, confocal microscopy and immunoblots showed that the deglycosylated soluble ERAD substrate SUBEX‐C57Y was stabilized in the cytosol by PNG1‐C255A, even when the proteasome was not specifically blocked. Yeast and cucumber PNG1 physically interact with the proteasome shuttling factor Rad23 (Hou et al., 2020; Suzuki et al., 2001), and mouse PNG1 interacts with the C terminus of CDC48/p97 at the *trans* side (Li et al., 2006). This suggests that PNG1 acts downstream of CDC48. As a result, N‐glycosylated substrates are translocated through the central pore of the CDC48 machinery (Twomey et al., 2019), unfolded and deglycosylated by PNG1 upon exiting on the *trans* side of CDC48 before degradation by the proteasome (Figure 8). Further studies are required to confirm the role of PNG1 and the sequence of processing steps in the cytosol.

The involvement of a deglycosylation step in ERAD of misfolded glycoproteins in plants was confirmed by purifying the reconstituted mNeonGreen2 protein in the cytosol. The immunoblots showed a faster migrating band, which may be due to enzymatic deglycosylation or an impairment of N‐glycosylation. The conversion of asparagine to aspartate is characteristic of PNGase‐mediated deglycosylation (Huang & Suzuki, 2020) and rules out an impaired N‐glycosylation or inefficient targeting to the ER. The co‐expression of the mutant PNG1‐C255A variant did not result in an accumulation of glycosylated ERAD substrates carrying oligomannosidic N‐glycans. This finding may seem puzzling at first, but it can be explained by the presence of cytosolic endoglycanases (ENGases). These enzymes can cleave the chitobiose core, leaving only a single GlcNAc residue attached to the N‐glycosylation site. In mammalian cells, the activity of ENGase contributes to the dysregulation of the ERAD process in cells that lack PNGase activity (Huang et al., 2015). This leads to the generation of aggregation prone N‐GlcNAc‐carrying ERAD substrates. Arabidopsis has two ENGases (Fischl et al., 2011; Kimura et al., 2011) that may act on misfolded glycoproteins in the cytosol when PNGase activity is abolished. Uncharacterized putative orthologs of these enzymes are present in *N. benthamiana*.

The confocal microscopy data suggest that complete deglycosylation is necessary for efficient degradation, as the cytosolic SUBEX‐C57Y ERAD client is stabilized by PNG1‐C255A‐HA. The presence of larger N‐glycans or single GlcNAc residues attached to the protein may hinder entry into the proteasome and pose a similar obstacle to degradation as folded proteins (Li et al., 2024). Under proteasome inhibition, the presence of mutated PNG1‐C255A resulted in an additive effect on the accumulation of the deglycosylated ERAD substrate. This may be due to incomplete inhibition of deglycosylation and proteasomal degradation. It is also possible that mechanisms compensating for proteasome inhibition, which have been described for mammalian cells, are dependent on an active PNGase (Galeone et al., 2020; Tomlin et al., 2017).

Ubiquitination of structurally defective proteins is necessary for various molecular events during ERAD. This process enables the recognition and interaction with the CDC48 ATPase complex, entry of substrates into the central pore, substrate unfolding, and generates a signal for degradation by the proteasome (Li et al., 2024). The yeast CDC48 ATPase's proposed substrate threading mechanism requires substrate ubiquitination and unfolding of a single ubiquitin (Twomey et al., 2019). In mammalian cells, removal of ubiquitin by a highly active virus‐derived deubiquitinase led to the accumulation of deglycosylated ERAD substrates in the cytosol (Ernst et al., 2011). In contrast, the expression of a YOD1 deubiquitinase that is enzymatically inactive resulted in the accumulation of the same ERAD clients in the ER lumen. This indicates that ubiquitination has different effects on the coupling and decoupling of ERAD steps. No substantial differences in total SUBEX‐C57Y ubiquitin levels were observed when co‐expressing OTU1 or OTU2 variants. This suggests that OTU1 and OTU2 have highly specific activities toward certain linkages (Radjacommare et al., 2014; Zang et al., 2020) or clients and are involved in the removal of specific ubiquitin molecules. Alternatively, both enzymes may have an ERAD substrate‐independent role, such as deubiquitination of ERAD components to regulate their activity (Baldridge & Rapoport, 2016; Bernardi et al., 2013; Chen et al., 2016). Co‐expression of OTU1‐C92S resulted in a slight but significant accumulation of the glycosylated SUBEX‐C57Y in the ER. This finding is similar to that of the non‐glycosylated membrane‐bound ERAD substrate MLO‐1, which strongly accumulated when an OTU1‐C92S variant was co‐expressed in *N. benthamiana* (Zang et al., 2020).

Several ubiquitin‐binding proteins, shuttling factors, chaperones, and cytosolic ubiquitin ligases participate in the degradation of substrates in mammalian cells and yeast (Fujisawa et al., 2022; Hu et al., 2020; Li et al., 2024). While putative orthologs of these proteins are present in plants, their role in the degradation process of retrotranslocated ERAD substrates has yet to be established. Recent findings with purified yeast complexes suggest that the proteasome and CDC48 compete for ubiquitinated substrates, with bidirectional substrate shuttling between the complexes (Li et al., 2024). Our data demonstrate that the identified ERAD steps are specific not only to SUBEX‐C57Y, but also to the soluble glycosylated ERAD substrate NBRI1‐5. This pathway may represent the default pathway for this type of substrate. It remains to be determined whether membrane‐anchored, topologically diverse ERAD and more compactly folded substrates undergo the same series of events for their degradation (Shin et al., 2018; Vitali et al., 2024). In summary, this study has revealed new steps in the degradation of misfolded glycoproteins in plants. Further research is necessary to define the specific steps and molecular players required for ERAD of misfolded glycoproteins in the cytosol.

## EXPERIMENTAL PROCEDURES

### Plant material

*Arabidopsis thaliana* plants were grown under long‐day conditions (16 h light/8 h dark) at 22°C. The *hrd1a hrd1b* double knockout mutant was obtained by crossing the T‐DNA insertion lines *hrd1a* (SALK_061776) and *hrd1b* (SALK_032914) as described previously (Su et al., 2011). The Col‐0 wild‐type line expressing SUBEX‐C57Y‐GFP (p47‐SUBEX‐C57Y) was previously generated (Hüttner, Veit, Vavra, Schoberer, Dicker, et al., 2014). For inhibitor treatment, 12‐day‐old *A. thaliana* seedlings were incubated for 24 h in 0.5× MS medium supplemented with 1% (w/v) sucrose and 50 μm kifunensine (Santa Cruz Biotechnology, Dallas, TX, USA) or 20 μm bortezomib (BTZ; Sigma‐Aldrich, Vienna, Austria). *N. benthamiana* was grown on soil under long‐day conditions at 24°C. For inhibitor treatments in *N. benthamiana*, 20 μm BTZ, 50 μm kifunensine, and/or the indicated concentration of the proteasome inhibitor MG132 (Sigma‐Aldrich) were infiltrated into leaves before harvesting of the material for protein extraction. For endoglycosidase digestion of glycoproteins, transient expression was carried out in the ΔXT/FT *N. benthamiana* line which mainly produces glycoproteins with complex N‐glycans that are sensitive to PNGase F digestion due to the absence of core fucose residues (Strasser et al., 2008).

### Plasmid constructs

The vector p47‐SUBEX‐C57Y for expression of SUBEX‐C57Y‐GFP (endogenous signal peptide and misfolded ectodomain of *A. thaliana* STRUBBELIG‐*At1g11130*), the vector p47‐SUBEX‐WT for SUBEX‐WT‐GFP (endogenous signal peptide and non‐mutated ectodomain domain from STRUBBELIG) expression, the vector p47‐SUBEX‐C57Y‐NQ123 for the expression of SUBEX‐C57Y‐NQ123‐GFP (endogenous signal peptide and mutated, non‐glycosylated SUBEX‐C57Y variant), p47‐MNS4 (MNS4‐GFP) (Hüttner, Veit, Vavra, Schoberer, Dicker, et al., 2014), p117‐SP‐mRFP‐SUBEX‐C57Y (SP‐RFP‐SUBEX‐C57Y, *A. thaliana* calnexin 1 signal peptide) (Shin et al., 2018), and p45‐AtCDC48A‐QQ (RFP‐CDC48‐QQ) (Shin, König‐Beihammer, et al., 2021) were described previously.

For the expression of the mNeonGreen2 fluorescent protein parts 1–10 (NG10) (Feng et al., 2017), a synthetic DNA fragment (GeneArt Gene Synthesis; Thermo Fisher Scientific, Vienna, Austria) that was codon‐optimized for *N. benthamiana* was cloned into the *Xba*I/*Bam*HI sites of the plant expression vector pPT2 (Strasser et al., 2005) to generate pPT2‐NG10. pPT2‐SP‐NG10 (SP‐NG10) was constructed in the same way using a synthetic DNA fragment encoding the barley α‐amylase signal peptide (Table S1). pPT2‐SP‐SUBEX‐C57Y‐NG11 (SP‐SUBEX‐C57Y‐NG11, endogenous STRUBBELIG signal peptide), pPT2‐SUBEX‐C57Y‐NG11 (SUBEX‐C57Y‐NG11, no signal peptide), p48‐NG11‐SUBEX‐C57Y (SP‐NG11‐SUBEX‐C57Y‐RFP, endogenous STRUBBELIG signal peptide), and p117‐SP‐RFP‐SUBEX‐C57Y‐NG11 (SP‐RFP‐SUBEX‐C57Y‐NG11, *A. thaliana* calnexin 1 signal peptide) were constructed from synthetic DNA fragments cloned into *Xba*I/*Bam*HI sites of plant expression vectors pPT2, p48, and p117 (Shin et al., 2018), respectively. For the constructs p117‐SP‐SUBEX‐C57Y‐NQ123‐NG11 (expression of SP‐RFP‐SUBEX‐C57Y‐NQ123‐NG11) and p117‐SP‐SUBEX‐WT‐NG11 (expression of SP‐RFP‐SUBEX‐WT‐NG11), PCR fragments obtained by amplification of p47‐SUBEX‐C57Y‐NQ123 or p47‐SUBEX‐WT (Hüttner, Veit, Vavra, Schoberer, Liebminger, et al., 2014) with primers SUB_F and SUB_R (Table S2) were cloned into *Xba*I/*Bam*HI digested p117‐SP‐RFP‐SUBEX‐C57Y‐NG11, which replaces the SUBEX‐C57Y sequence.

A synthetic DNA fragment consisting of the *UBQ10* promoter, the YFP sequence with a C‐terminal myc tag and the g7 terminator from pPT2 was synthesized (GeneArt Gene Synthesis) and ligated into the *Kpn*I site of p47‐SUBEX‐C57Y to generate p74‐SUBEX‐C57Y. This vector was used in co‐expression experiments to quantify SUBEX‐C57Y degradation. YFP expression was used to normalize for variations in expression in individual plants.

The GFP‐CDC48‐QQ (CDC48A: *At3g09840*) expression vector p37‐AtCDC48A‐QQ was generated as previously described for p45‐AtCDC48A‐QQ (Shin, König‐Beihammer, et al., 2021) by replacing the RFP sequence with GFP. GFP‐CDC48 and RFP‐CDC48 expression vectors p37‐AtCDC48A and p45‐AtCDC48A, respectively, were cloned in the same way as described previously for the QQ variants (Shin, König‐Beihammer, et al., 2021) using the K482 plasmid provided by Ralph Panstruga (RWTH Aachen, University, Aachen, Germany) (Müller et al., 2005). For HRD1A expression, the full‐length coding sequence of HRD1A (*At3g16090*) was amplified from *A. thaliana* cDNA using the primers At3g16090_F and At3g16090_R, *Xba*I/*Bam*HI digested and cloned into *Xba*I/*Bam*HI sites of p41 (HA tag) (Shin et al., 2017) or p47 (GFP tag). The catalytically defective HRD1A variant HRD1A‐C329S was generated by site‐directed mutagenesis using At3g16090_MF/At3g16090_MR primers and the QuikChange II site‐directed mutagenesis protocol (Agilent, Santa Clara, CA, USA). For RFP‐SEL1L expression the Arabidopsis *SEL1L* (*At1g18260*) coding sequence was amplified by PCR from p60SEL1L plasmid (Hüttner et al., 2012) with primers At1g18260_F/At1g18260_R. The PCR product was *Spe*I digested and cloned into *Xba*I digested expression vector p110 (Göritzer et al., 2020). The RFP‐OS9 expression vector p110‐OS9 was created by amplification of the Arabidopsis *OS9* (*At5g35080*) coding sequence from plasmid p31‐AtOS9‐mRFP (Hüttner et al., 2012) using primers At5g35080_F/At5g35080_R. The PCR product was *Xba*I/*Bam*HI digested and cloned into *Xba*I/*Bam*HI sites of p110.

For OTU2 expression, the full‐length OTU2 (*At1g50670*) coding sequence was amplified from *A. thaliana* cDNA using the primers OTU2_F and OTU2_R, *Xba*I/*Bam*HI digested and cloned into *Xba*I/*Bam*HI sites of p45 (N‐terminal RFP tag). The catalytically defective OTU2 variant OTU2‐C16S was obtained by gene synthesis (GeneArt Gene Synthesis) and OTU2‐C63S was generated by site‐directed mutagenesis using OTU2_MF/OTU2_MR primers. For OTU1 expression, the full‐length coding sequence of OTU1 (*At1g28120*) was amplified from *A. thaliana* cDNA using the primers OTU1_F and OTU1_R, *Xba*I/*Bam*HI digested and cloned into *Xba*I/*Bam*HI sites of p45 (N‐terminal RFP tag), amplified with OTU1_XF and OTU1_XR and cloned into p48 (C‐terminal RFP tag) or amplified with OTU1_XF and OTU1_R and cloned into pPT2 (no tag). The catalytically defective OTU1 variant OTU1‐C92S, cloned into p45 (p45‐OTU1_C92S) or p48 (p48‐OTU1‐C92S), was obtained by gene synthesis (Table S1). The catalytically defective PNG1 variant PNG1‐C255A and the non‐mutated PNG1 variant were obtained by gene synthesis (GeneArt Gene Synthesis) of an open reading frame encoding the putative *N. tabacum* PNG1 (UniProt: A0A1S3Z191). The synthetic DNA fragments were cloned into *Xba*I/*Bam*HI sites of p41 to generate p41‐PNG1‐C255A (PNG1‐C255A‐HA) and p41‐PNG1 (PNG1‐HA) and p48 to generate p48‐PNG1‐C255A (PNG1‐C255A‐RFP) and p48‐PNG1 (PNG1‐RFP). To construct the vector p117‐SP‐NBRI1‐5‐NG11 (SP‐RFP‐NBRI1‐5, *A. thaliana* calnexin 1 signal peptide), the DNA region coding for the N‐terminal BRI1 (*At4g39400*) amino acids 24–212 was amplified from *bri1‐5* cDNA (Shin et al., 2018) using primers BRI1_F and BRI1_R, *Spe*I/*Bam*HI digested and cloned into *Xba*I/*Bam*HI digested p117‐SP‐RFP‐SUBEX‐C57Y‐NG11 to replace the SUBEX‐C57Y coding sequence with the one for NBRI1‐5. In the p41, p47, p48, p110, and p117 expression vectors, expression is under the control of the *A. thaliana UBQ10* promoter. In the pPT2, p37, and p45 expression vectors, expression is under the control of the *CaMV35S* promoter.

### Confocal microscopy

For subcellular localization studies, Agrobacteria carrying one of the different expression constructs were diluted to an OD_600_ of 0.1 or 0.2, mixed together and infiltrated into in leaves of 5‐week‐old *N. benthamiana* plants as described previously (Schoberer et al., 2009). Sections of infiltrated leaves were analyzed 48 h after infiltration on a Leica TCS SP5 confocal microscope as previously described in detail (Schoberer et al., 2014).

### Purification of SUBEX‐C57Y‐GFP

SUBEX‐C57Y‐GFP was purified from *A. thaliana* seedlings using GFP‐Trap® agarose (Chromotek, Planegg‐Martinsried, Germany). Briefly, frozen seedlings were homogenized using a mixer mill (Retsch, Haan, Germany) with steel grinding balls, the homogenized material was resuspended in 1× PBS. After centrifugation, the clarified supernatant was incubated with GFP‐Trap® agarose (Chromotek) as described previously (Hüttner, Veit, Vavra, Schoberer, Dicker, et al., 2014). Proteins were eluted and subjected to SDS‐PAGE and immunoblotting.

### Co‐purification of transiently expressed ERAD components

Leaves of 5‐week‐old *N. benthamiana* plants were infiltrated with a mixture of Agrobacteria (OD_600_ of 0.1–0.2) carrying the indicated expression constructs. Co‐purification with GFP‐Trap®, RFP‐Trap®, or mNeonGreen‐Trap® agarose (Chromotek) and immunoblotting were performed as previously described in detail (Hüttner, Veit, Vavra, Schoberer, Dicker, et al., 2014). Primary antibodies against GFP/YFP (Roche, Vienna, Austria), RFP (Chromotek), HA (Roche), α‐tubulin (Sigma‐Aldrich), and ubiquitin (P4D1; Santa Cruz Biotechnology) were commercially available. For purifications involving the subsequent detection of ubiquitin, the GFP‐Trap® or mNeonGreen‐Trap® agarose was incubated in extraction buffer supplemented with 1% protease inhibitor cocktail (P9599; Sigma‐Aldrich), 50 μm MG132 (Sigma‐Aldrich), and 10 mm *N*‐ethylmaleimide (Sigma‐Aldrich). For *in vitro* deglycosylation, purified proteins were incubated with endoglycosidase H (EndoH) or peptide‐*N*‐glycosidase F (PNGase F) (New England Biolabs, Frankfurt am Main, Germany) according to the manufacturer's instructions.

### MS analysis of peptides

Leaves of 5‐week‐old *N. benthamiana* were infiltrated with a mixture of Agrobacteria (OD_600_ of 0.1) carrying p117‐SP‐RFP‐SUBEX‐C57Y‐NG11 and Agrobacteria (OD_600_ of 0.1) carrying pPT2‐NG10. Twenty‐nine hours after infiltration of the Agrobacteria mixture, 20 μm BTZ was infiltrated into the same leaf. Forty‐eight hours after the infiltration of the Agrobacteria mixture, 250 mg of leaf material was harvested, frozen in liquid nitrogen, and homogenized using a mixer mill with steel grinding balls. Tris–HCl (500 μl, 10 mm, pH 7.5) supplemented with 150 mm NaCl, 0.5 mm EDTA, and 0.5% (v/v) NP40 (Sigma‐Aldrich) was added and after centrifugation steps, the clear supernatant was incubated with 10 μl mNeonGreen‐Trap® agarose (Chromotek) for 1 h at 4°C. After centrifugation and washing with 10 mm Tris‐HCl pH 7.5, 150 mm NaCl, 0.5 mm EDTA, 0.025% (v/v) NP40, the beads were incubated in 1× Laemmli sample buffer at 95°C for 5 min to elute the bound proteins. The eluate was separated by SDS‐PAGE and the area migrating at the position of the 70 kDa marker band was excised from the gel, S‐alkylated with iodoacetamide, and trypsin (Promega) digested as described previously (Stadlmann et al., 2008).

The digested samples were loaded on a nanoEase C18 column (nanoEase M/Z HSS T3 Column, 100 Å, 1.8 μm, 300 μm × 150 mm; Waters, Milford, MA, USA) using 0.1% (v/v) formic acid as the aqueous solvent. A gradient from 1% B (B: 80% acetonitrile, 0.1% formic acid) to 40% B in 50 min was applied, followed by a 10‐min gradient from 40% B to 95% B that facilitates elution of large peptides, at a flow rate of 6 μl min^−1^. Detection was performed with an Orbitrap MS (Exploris 480; Thermo Fisher Scientific) equipped with the standard H‐ESI source in positive ion, DDA mode (=switching to MS/MS mode for eluting peaks). MS scans were recorded (range: 350–3200 Da) and the 20 highest peaks were selected for fragmentation. Instrument calibration was performed using Pierce FlexMix Calibration Solution (Thermo Fisher Scientific). The analysis files were analyzed manually and the respective deamidated peptides were identified by MS/MS.

### Statistical analyses

Data are represented by bar charts showing the mean ± SD of all data points. The numbers of replicates (*n*) are given in each figure legend. Statistical analysis (two tailed Student's *t* test) was performed using GraphPad Prism version 10.0.1.

### ACCESSION NUMBERS

The Arabidopsis AGI locus identifiers of genes used in this article are BRI1 (*At4g39400*), CDC48A (*At3g09840*), HRD1A (*At3g16090*), OTU1 (*At1g28120*), OTU2 (*At1g50670*), PNG1 (*At5G49570*), SEL1L (*At1g18260*), STRUBBELIG (*At1g11130*), and OS9 (*At5g35080*). The amino acid sequence for *N. tabacum* PNG1 can be found under this UniProt accession number: A0A1S3Z191.

## CONFLICT OF INTEREST

The authors declare no competing interests.

## Supporting information


**Figure S1.** EndoH digestion results in a shift in mobility of the faster migrating band detected upon accumulation of SP‐RFP‐SUBEX‐C57Y‐NG11.
**Figure S2.** Kifunensine treatment prevents retrotranslocation of SP‐RFP‐SUBEX‐C57Y‐NG11.
**Figure S3.** mNeonGreen2 is reconstituted when SP‐RFP‐SUBEX‐C57Y‐NQ123‐NG11 or SP‐RFP‐WT‐NG11 are co‐expressed with ER‐targeted SP‐NG10.
**Figure S4.** mNeonGreen2 is reconstituted when SP‐NG11‐SUBEX‐C57Y‐RFP is co‐expressed with SP‐NG10.
**Figure S5.** HRD1A interacts with SEL1L.
**Figure S6.** mNeonGreen2 is not reconstituted when SP‐RFP‐SUBEX‐C57Y‐NG11 is co‐expressed with NG10 and HRD1A‐C329S‐HA.
**Figure S7.** mNeonGreen2 is not reconstituted when SP‐RFP‐SUBEX‐C57Y‐NG11 is co‐expressed with NG10 and RFP‐CDC48‐QQ.
**Figure S8.** A block of SP‐RFP‐SUBEX‐C57Y degradation by HRD1A‐C329S‐GFP co‐expression prevents ubiquitination.
**Figure S9.** mNeonGreen2 is not reconstituted when SP‐RFP‐SUBEX‐C57Y‐NG11 and NG10 are co‐expressed with RFP‐OTU1 or RFP‐OTU2.
**Figure S10.** RFP‐OTU2‐C63S co‐expression does not affect the mNeonGreen2 reconstitution.
**Figure S11.** OTU1 or OTU1‐RFP expression does not affect ubiquitination of SP‐SUBEX‐C57Y‐GFP after block of the degradation by RFP‐CDC48‐QQ co‐expression.
**Figure S12.** LC–MS/MS analysis of the deglycosylated SUBEX‐C57Y peptide WQGVVCDSSNITEIR.
**Figure S13.** PNG1‐C255A‐RFP co‐expression leads to accumulation of the mNeonGreen2 signal in the cytosol.
**Figure S14.** PNG1 co‐expression does not lead to accumulation of the mNeonGreen2 signal in the cytosol or nucleus.
**Figure S15.** No SP‐RFP‐SUBEX‐C57Y‐NG11 is purified with NG‐trap beads when the degradation is blocked by RFP‐CDC48‐QQ co‐expression.
**Figure S16.** The N‐glycosylated ERAD substrate SP‐RFP‐NBRI1‐5‐NG11 is retrotranslocated and degraded by the proteasome.
**Figure S17.** Retrotranslocation of the N‐glycosylated ERAD substrate SP‐RFP‐NBRI1‐5‐NG11 is blocked by HRD1A‐C329S co‐expression.
**Figure S18.** PNG1‐C255A‐HA blocks the degradation of the retrotranslocated N‐glycosylated ERAD substrate SP‐RFP‐NBRI1‐5‐NG11.
**Table S1.** List of synthetic DNA sequences used in this study.
**Table S2.** List of primers used in this study.

## Data Availability

The data that support the findings of this study are available from the corresponding author upon reasonable request.
