# Supplementary material for: Elucidation of the late steps in the glycan‐dependent ERAD of soluble misfolded glycoproteins
Source: Plant J. 2024 Dec 6;121(1):e17185. doi: 10.1111/tpj.17185 (PMC11712024; doi:10.1111/tpj.17185)
Supplement: Supplementary file 1 — Figure S1. EndoH digestion results in a shift in mobility of the faster migrating band detected upon accumulation of SP‐RFP‐SUBEX‐C57Y‐NG11. Figure S2. Kifunensine treatment prevents retrotranslocation of SP‐RFP‐SUBEX‐C57Y‐NG11. Figure S3. mNeonGreen2 is reconstituted when SP‐RFP‐SUBEX‐C57Y‐NQ123‐NG11 or SP‐RFP‐WT‐NG11 are co‐expressed with ER‐targeted SP‐NG10. Figure S4. mNeonGreen2 is reconstituted when SP‐NG11‐SUBEX‐C57Y‐RFP is co‐expressed with SP‐NG10. Figure S5. HRD1A interacts with SEL1L. Figure S6. mNeonGreen2 is not reconstituted when SP‐RFP‐SUBEX‐C57Y‐NG11 is co‐expressed with NG10 and HRD1A‐C329S‐HA. Figure S7. mNeonGreen2 is not reconstituted when SP‐RFP‐SUBEX‐C57Y‐NG11 is co‐expressed with NG10 and RFP‐CDC48‐QQ. Figure S8. A block of SP‐RFP‐SUBEX‐C57Y degradation by HRD1A‐C329S‐GFP co‐expression prevents ubiquitination. Figure S9. mNeonGreen2 is not reconstituted when SP‐RFP‐SUBEX‐C57Y‐NG11 and NG10 are co‐expressed with RFP‐OTU1 or RFP‐OTU2. Figure S10. RFP‐OTU2‐C63S co‐expression does not affect the mNeonGreen2 reconstitution. Figure S11. OTU1 or OTU1‐RFP expression does not affect ubiquitination of SP‐SUBEX‐C57Y‐GFP after block of the degradation by RFP‐CDC48‐QQ co‐expression. Figure S12. LC–MS/MS analysis of the deglycosylated SUBEX‐C57Y peptide WQGVVCDSSNITEIR. Figure S13. PNG1‐C255A‐RFP co‐expression leads to accumulation of the mNeonGreen2 signal in the cytosol. Figure S14. PNG1 co‐expression does not lead to accumulation of the mNeonGreen2 signal in the cytosol or nucleus. Figure S15. No SP‐RFP‐SUBEX‐C57Y‐NG11 is purified with NG‐trap beads when the degradation is blocked by RFP‐CDC48‐QQ co‐expression. Figure S16. The N‐glycosylated ERAD substrate SP‐RFP‐NBRI1‐5‐NG11 is retrotranslocated and degraded by the proteasome. Figure S17. Retrotranslocation of the N‐glycosylated ERAD substrate SP‐RFP‐NBRI1‐5‐NG11 is blocked by HRD1A‐C329S co‐expression. Figure S18. PNG1‐C255A‐HA blocks the degradation of the retrotranslocated N‐glycosylated ERAD substra [file TPJ-121-0-s001.pdf]

Supplemental data: Schoberer et al. (2024) Elucidation of the late steps in the glycan-dependent ERAD of soluble misfolded glycoproteins

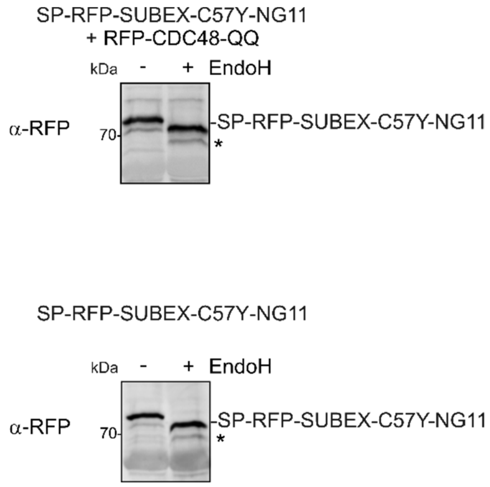

Supplemental Figure S1. EndoH digestion results in a shift in mobility of the faster migrating band detected upon accumulation of SP-RFP-SUBEX-C57Y-NG11. SP-RFP-SUBEX-C57Y-NG11 was transiently co-expressed in *N. benthamiana* leaves without or with RFP-CDC48-QQ. Protein extracts were subjected to digestion by endoglycosidase H (EndoH) which removes oligomannosidic N-glycans. The faster migrating band that shifts due to the deglycosylation is marked by an asterisk.

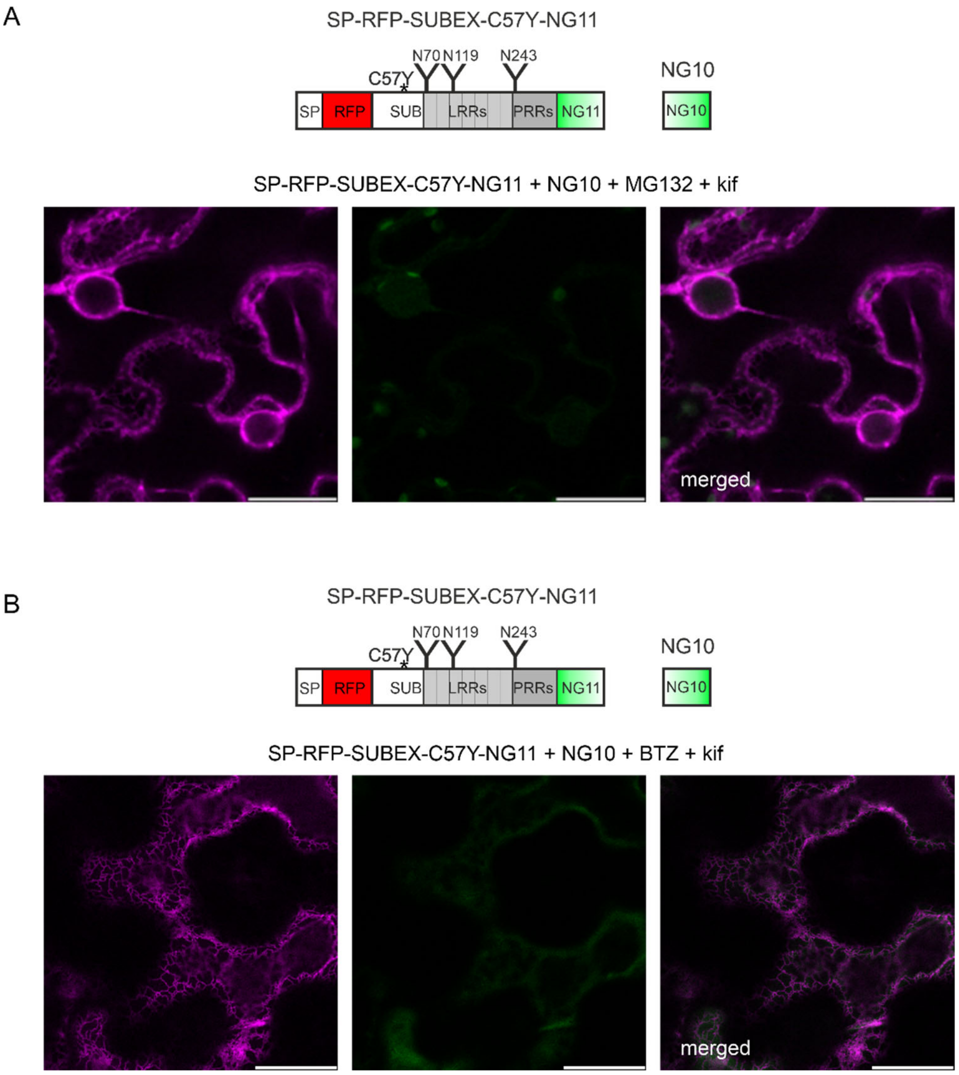

Supplemental Figure S2. Kifunensine treatment prevents retrotranslocation of SP-RFP-SUBEX-C57Y-NG11. (**A**) Representative confocal images of SP-RFP-SUBEX-C57Y-NG11 co-expressed with NG10 in *N. benthamiana* leaf epidermal cell in the presence of 40 µM MG132 and 50 µM kifunensine (kif). (**B**) Representative confocal images of SP-RFP-SUBEX-C57Y-NG11 co-expressed with NG10 in *N. benthamiana* leaf epidermal cell in the presence of 20 µM bortezomib (BTZ) and 50 µM kifunensine (kif). MG132 or BTZ was co-infiltrated in order to prevent the possible degradation of the SUBEX-C57Y fusion protein in the cytosol. Scale bars = 10 µm.

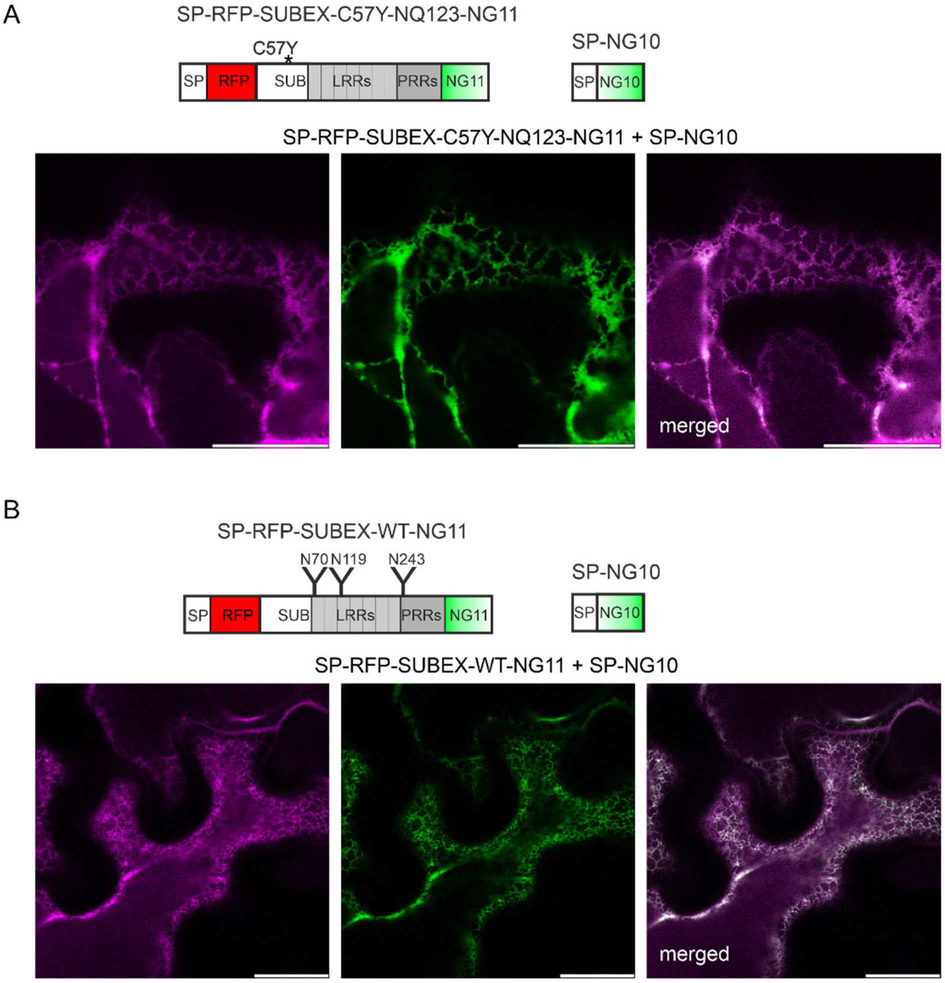

Supplemental Figure S3. mNeonGreen2 is reconstituted when SP-RFP-SUBEX-C57Y-NQ123-NG11 or SP-RFP-WT-NG11 are co-expressed with ER-targeted SP-NG10. (**A** and **B**) Representative confocal images showing RFP and mNeonGreen2 signals in the ER. To show that the expressed SP-RFP-SUBEX-C57Y-NQ123-NG11 or SP-RFP-SUBEX-WT-NG11 variants are competent for mNeonGreen2 reconstitution, they were co-expressed in *N. benthamiana* leaf epidermal cells with the ER-targeted SP-NG10. Scale bars = 10 µm.

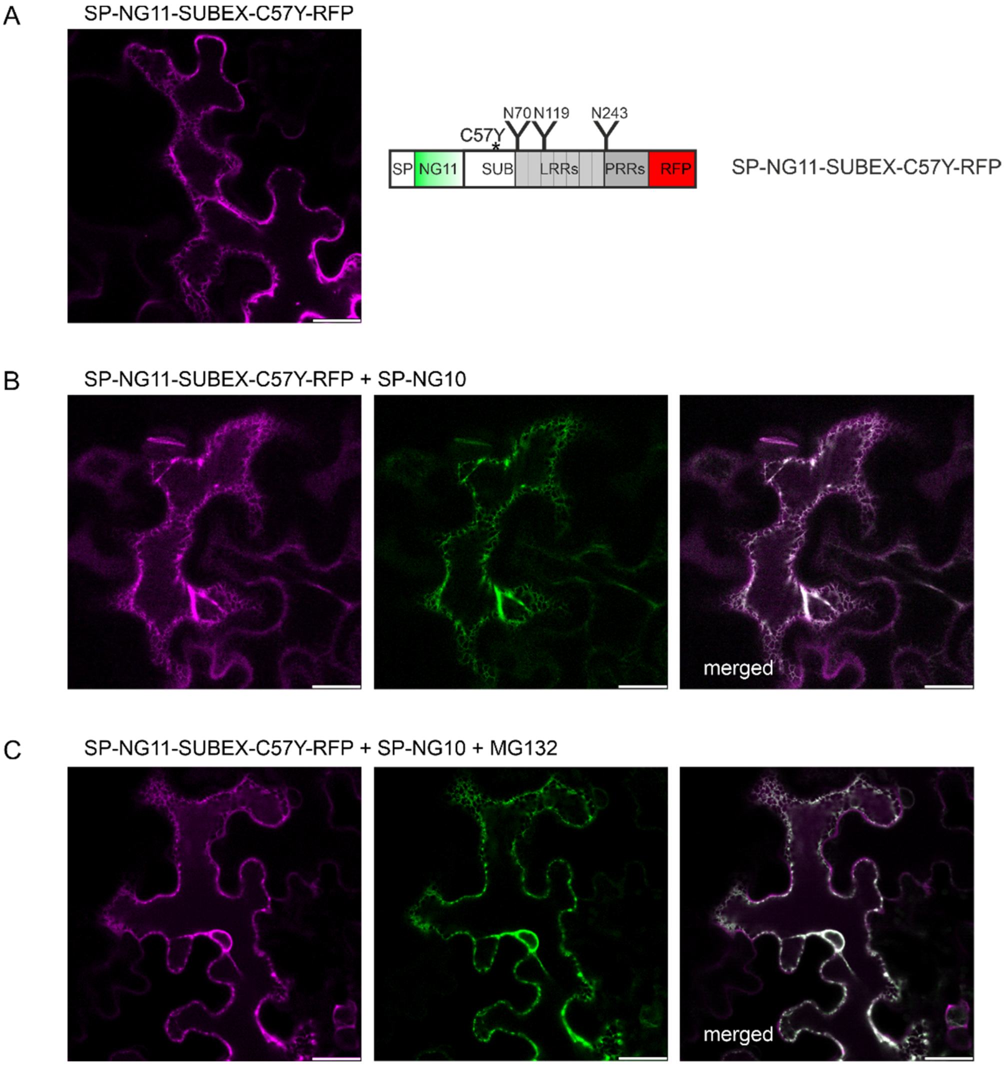

Supplemental Figure S4. mNeonGreen2 is reconstituted when SP-NG11-SUBEX-C57Y-RFP is co-expressed with SP-NG10. (**A** to **C**) Representative confocal images of SP-NG11-SUBEX-C57Y-RFP expressed in *N. benthamiana* leaf epidermal cells either alone (**A**), or co-expressed with SP-NG10 (**B**), or co-expressed with SP-NG10 in the presence of 40 µM MG132 (**C**). Scale bars = 10 µm.

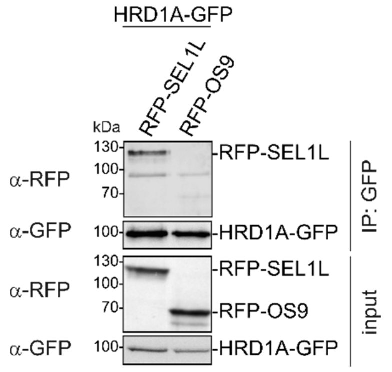

Supplemental Figure S5. HRD1A interacts with SEL1L. HRD1A-GFP was transiently co-expressed with either RFP-SEL1L or RFP-OS9 in *N. benthamiana* leaves. HRD1A-GFP was purified using GFP-Trap beads and co-purified proteins were analysed by immunoblotting with anti-RFP antibodies.

A

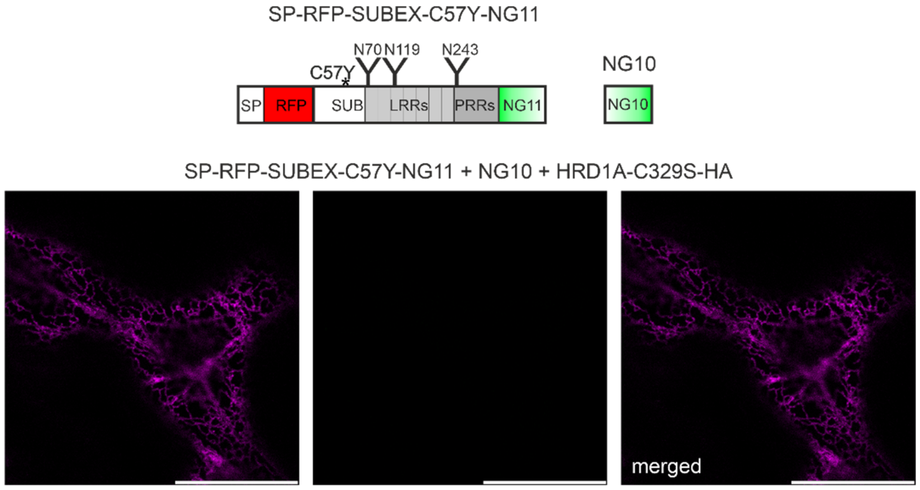

B

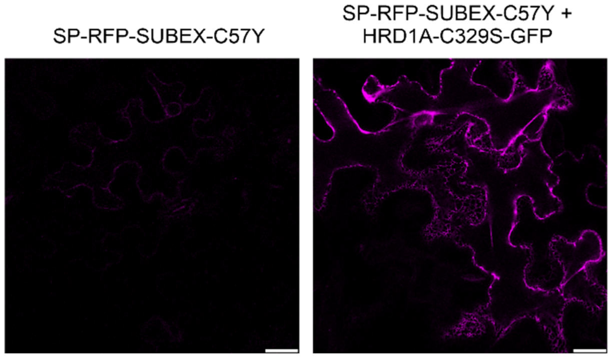

Supplemental Figure S6. mNeonGreen2 is not reconstituted when SP-RFP-SUBEX-C57Y-NG11 is co-expressed with NG10 and HRD1A-C329S-HA. Representative confocal images of *N. benthamiana* leaf epidermal cells expressing (**A**) SP-RFP-SUBEX-C57Y-NG11, NG10 and HRD1A-C329S-HA or (**B**) SP-RFP-SUBEX-C57Y without and with HRD1A-C329S-GFP are shown. Scale bars = 10 µm.

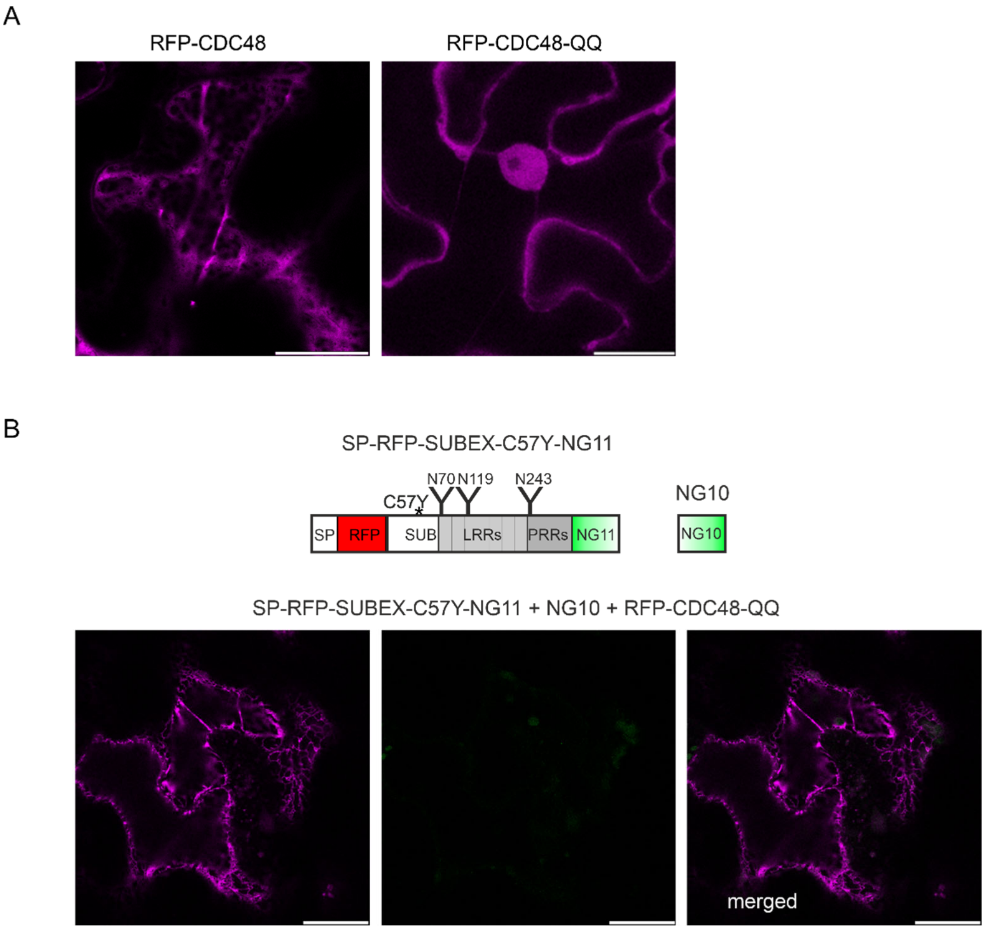

Supplemental Figure S7. mNeonGreen2 is not reconstituted when SP-RFP-SUBEX-C57Y-NG11 is co-expressed with NG10 and RFP-CDC48-QQ. (**A**) Representative confocal images of RFP-CDC48 and RFP-CDC48-QQ transiently expressed in *N. benthamiana* leaf epidermal cells. (**B**) Representative confocal images of SP-RFP-SUBEX-C57Y-NG11 co-expressed in *N. benthamiana* leaf epidermal cells with NG10 and RFP-CDC48-QQ. Scale bars = 10 µm.

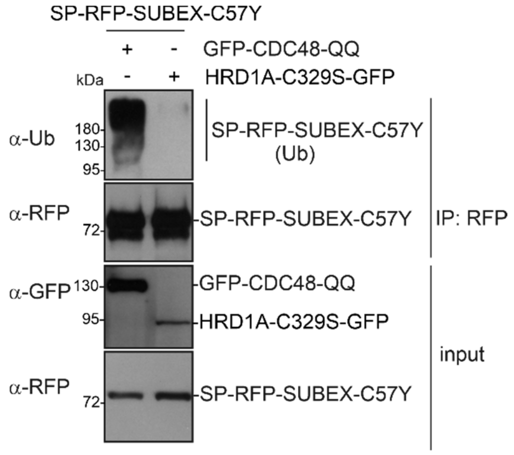

Supplemental Figure S8. A block of SP-RFP-SUBEX-C57Y degradation by HRD1A-C329S-GFP co-expression prevents ubiquitination. SP-RFP-SUBEX-C57Y was transiently co-expressed in *N. benthamiana* leaves with either GFP-CDC48-QQ or HRD1A-C329S-GFP, purified by binding to RFP-trap beads and subjected to immunoblotting with GFP, RFP and anti-ubiquitin (Ub) antibodies.

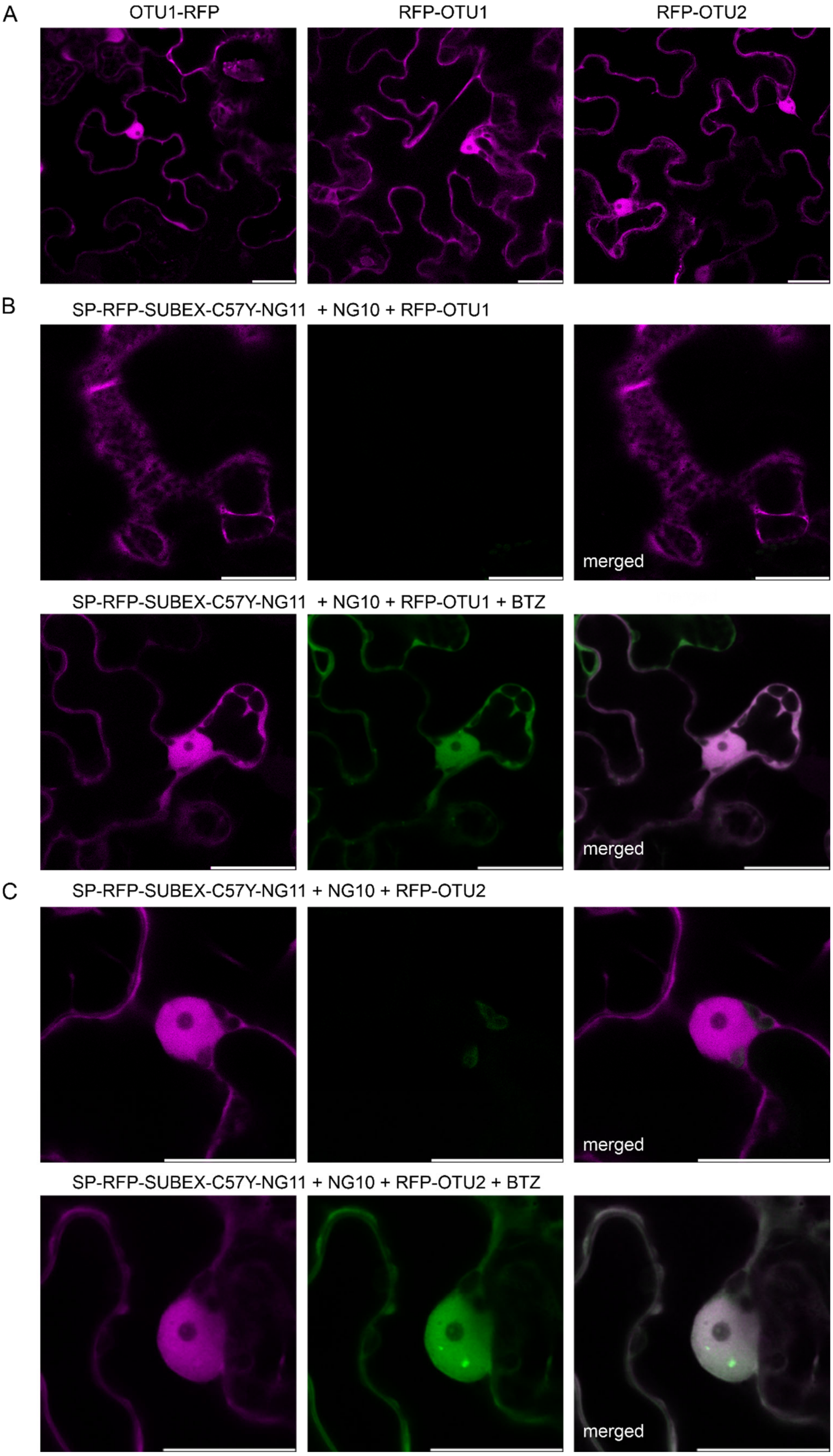
A
OTU1-RFP
RFP-OTU1
RFP-OTU2
B
SP-RFP-SUBEX-C57Y-NG11 + NG10 + RFP-OTU1
merged
SP-RFP-SUBEX-C57Y-NG11 + NG10 + RFP-OTU1 + BTZ
merged
C
SP-RFP-SUBEX-C57Y-NG11 + NG10 + RFP-OTU2
merged
SP-RFP-SUBEX-C57Y-NG11 + NG10 + RFP-OTU2 + BTZ
merged

Supplemental Figure S9. mNeonGreen2 is not reconstituted when SP-RFP-SUBEX-C57Y-NG11 and NG10 are co-expressed with RFP-OTU1 or RFP-OTU2. (**A**) Subcellular localization of OTU1-RFP, RFP-OTU1 and RFP-OTU2 variants. Representative confocal images are shown. The indicated fluorescent fusion proteins were transiently expressed in *N. benthamiana* leaf epidermal cells. (**B**) Representative confocal images of SP-RFP-SUBEX-C57Y-NG11 co-expressed in *N. benthamiana* leaf epidermal cells with NG10 and RFP-OTU1 in the absence or presence of 20 µM BTZ. (**C**) Representative confocal images of SP-RFP-SUBEX-C57Y-NG11 co-expressed with NG10 and RFP-OTU2 in the absence or presence of 20 µM BTZ. Scale bars = 10 µm.

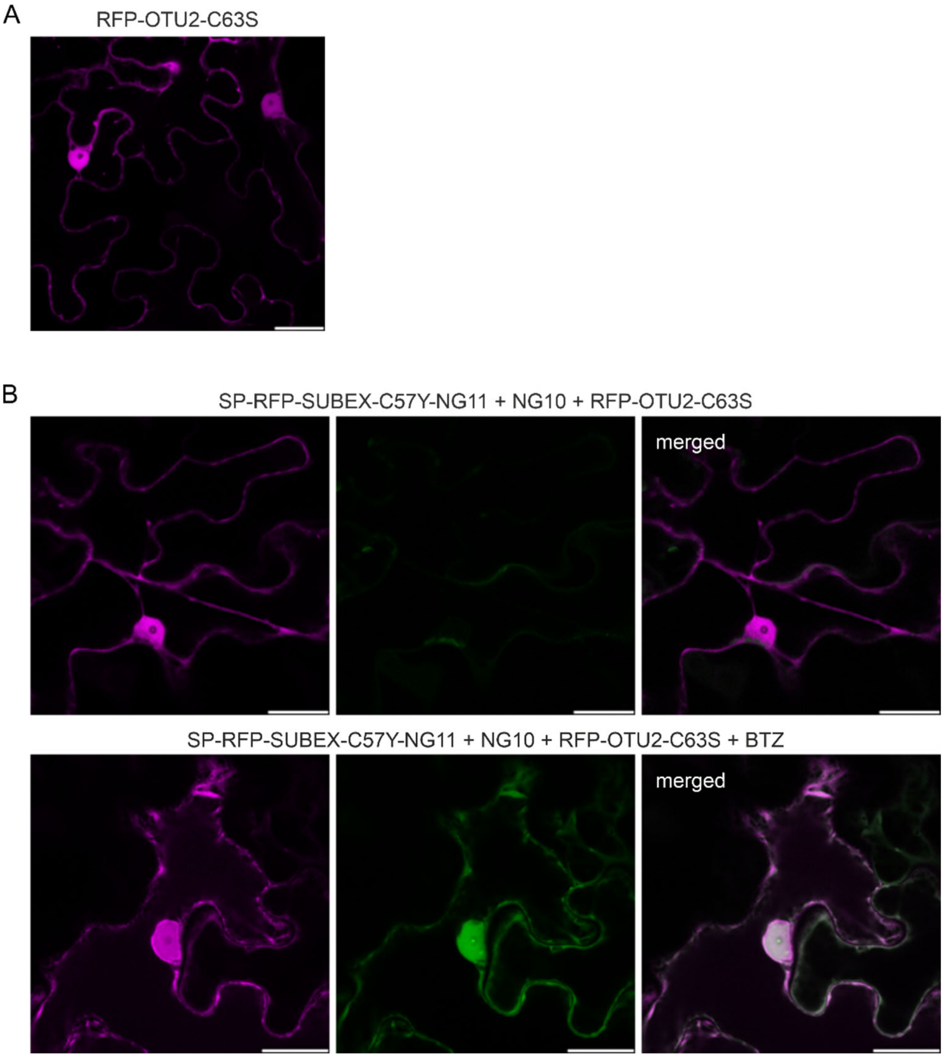

Supplemental Figure S10. RFP-OTU2-C63S co-expression does not affect the mNeonGreen2 reconstitution. (**A**) Subcellular localization of RFP-OTU2-C63S transiently expressed in *N. benthamiana* leaf epidermal cells. (**B**) Representative confocal images of SP-RFP-SUBEX-C57Y-NG11 co-expressed in *N. benthamiana* leaf epidermal cells with NG10 and RFP-OTU2-C63S in the absence or presence of 20 µM BTZ. Scale bars = 10 µm. In the absence of BTZ no specific mNeonGreen2 signal is detectable.

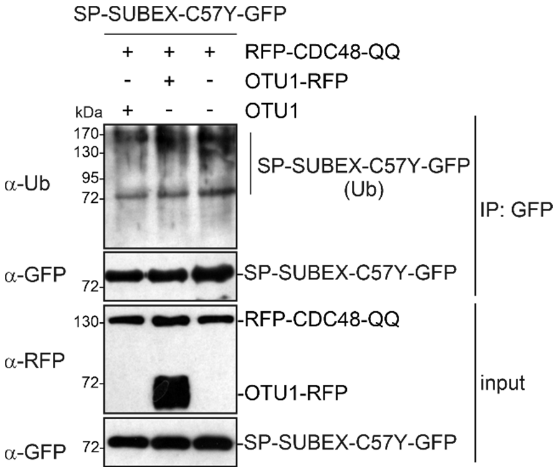

Supplemental Figure S11. OTU1 or OTU1-RFP expression does not affect ubiquitination of SP-SUBEX-C57Y-GFP after block of the degradation by RFP-CDC48-QQ co-expression. Tagged (OTU1-RFP) or untagged OTU1 were transiently co-expressed in *N. benthamiana* leaves with RFP-CDC48-QQ and SP-SUBEX-C57Y-GFP. SP-SUBEX-C57Y-GFP was purified by binding to GFP-trap beads and subjected to SDS-PAGE and immunoblotting with GFP, RFP and anti-ubiquitin (Ub) antibodies.

Fragment Ion Table, monoisotopic masses

Seq	#	B	Y	# (+1)
W	1	187.08663	1764.82070	15
Q	2	315.14521	1578.74139	14
G	3	372.16667	1450.68281	13
V	4	471.23509	1393.66135	12
V	5	570.30350	1294.59294	11
C	6	730.33269	1195.52452	10
D	7	845.35963	1035.49534	9
S	8	932.39166	920.46839	8
S	9	1019.42369	833.43637	7
D	10	1134.45063	746.40434	6
I	11	1247.53469	631.37739	5
T	12	1348.58237	518.29333	4
E	13	1477.62496	417.24565	3
I	14	1590.70903	288.20306	2
R	15	1746.81014	175.11900	1

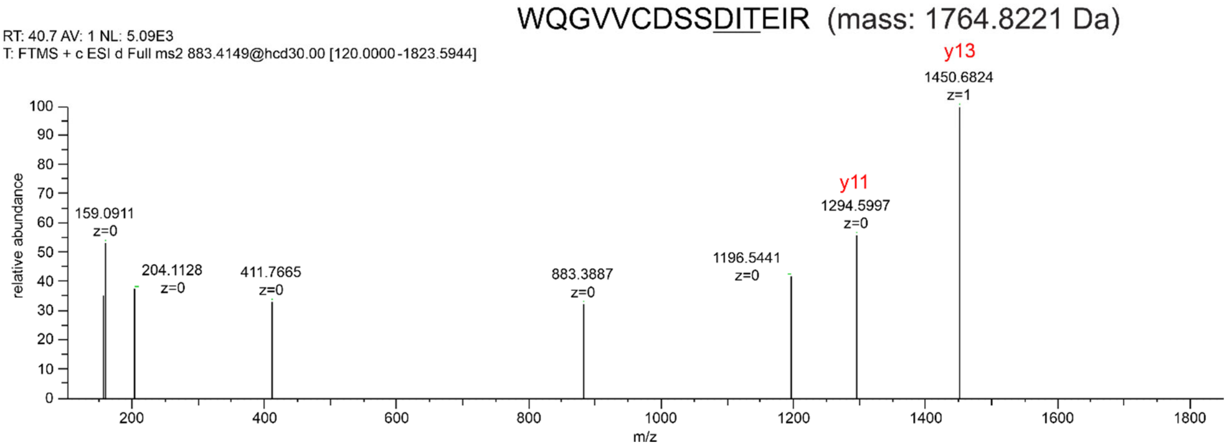

Supplemental Figure S12. LC-MS/MS analysis of the deglycosylated SUBEX-C57Y peptide WQGVVCDSSNITEIR. SP-RFP-SUBEX-C57Y-NG11 was transiently co-expressed with NG10 and BTZ (20 µM) in *N. benthamiana* leaves. The reconstituted fluorescent fusion protein was purified by binding to NG-trap beads, subjected to SDS-PAGE under reducing conditions and the 70 kDa band was excised, trypsin digested and analysed by LC-MS/MS. Due to the deglycosylation, the peptide WQGVVCDSS**N**ITEIR carrying the N-glycosylation site 1 of SUBEX-C57Y is converted to WQGVVCDSS**D**ITEIR. Masses for characteristic fragment ions are indicated (in red). The monoisotopic masses for the fragment ions were calculated using the Fragment Ion Calculator tool (http://db.systemsbiology.net:8080/proteomicsToolkit/FragIonServlet.html).

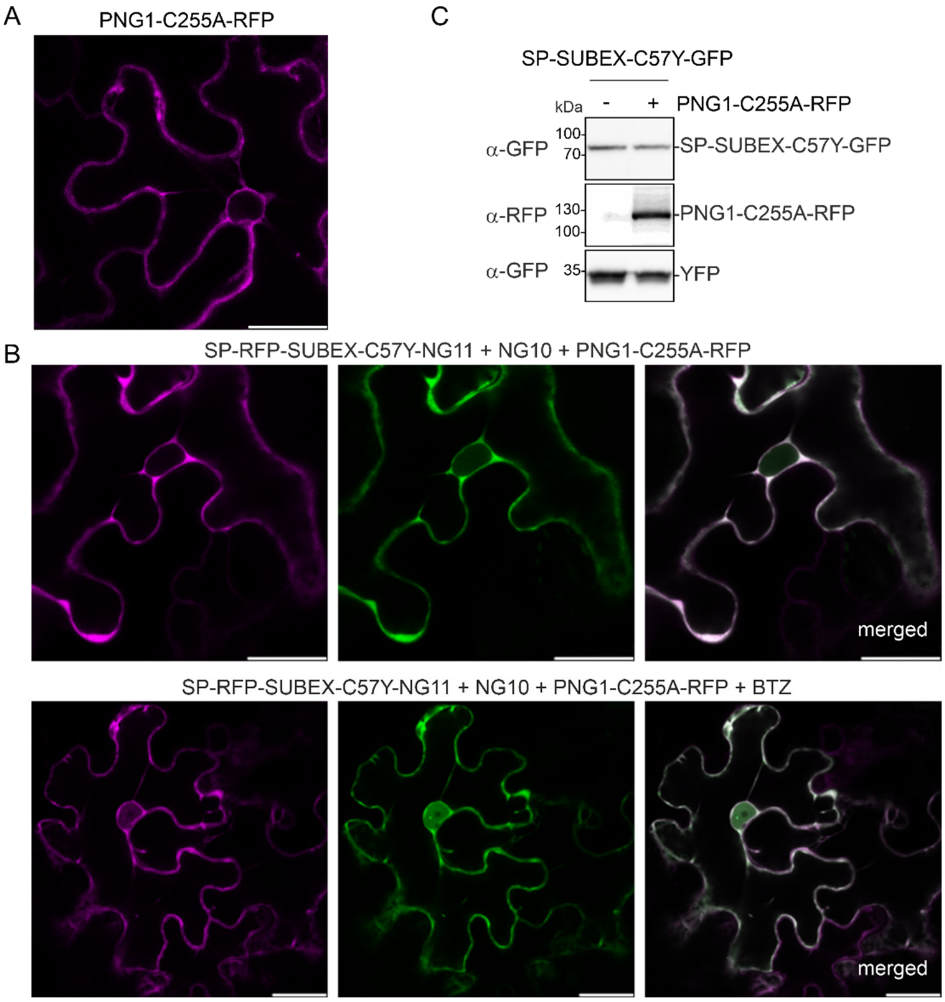

Supplemental Figure S13. PNG1-C255A-RFP co-expression leads to accumulation of the mNeonGreen2 signal in the cytosol. (**A**) PNG1-C255A-RFP was transiently expressed in *N. benthamiana* leaf epidermal cells. A representative confocal image is shown. (**B**) SP-RFP-SUBEX-C57Y-NG11 was transiently co-expressed in *N. benthamiana* leaf epidermal cells with NG10 and PNG1-C255A-RFP in the absence or presence of 20 µM BTZ. It is noteworthy that co-expression of PNG1-C255A-RFP results in the accumulation of the NG-tagged protein in the cytosol and nuclear envelope, with minimal or no labelling of the nucleoplasm. By contrast, inhibition of the proteasome by treatment with BTZ leads to accumulation of the NG-tagged protein in the cytosol, nuclear envelope and nucleoplasm. Scale bars = 10 µm. (**C**) Immunoblot analysis of SP-SUBEX-C57Y-GFP transiently co-expressed in *N. benthamiana* leaves with PNG1-C255A-RFP. Co-expressed YFP was used for normalization of the SP-SUBEX-C57Y-GFP expression.

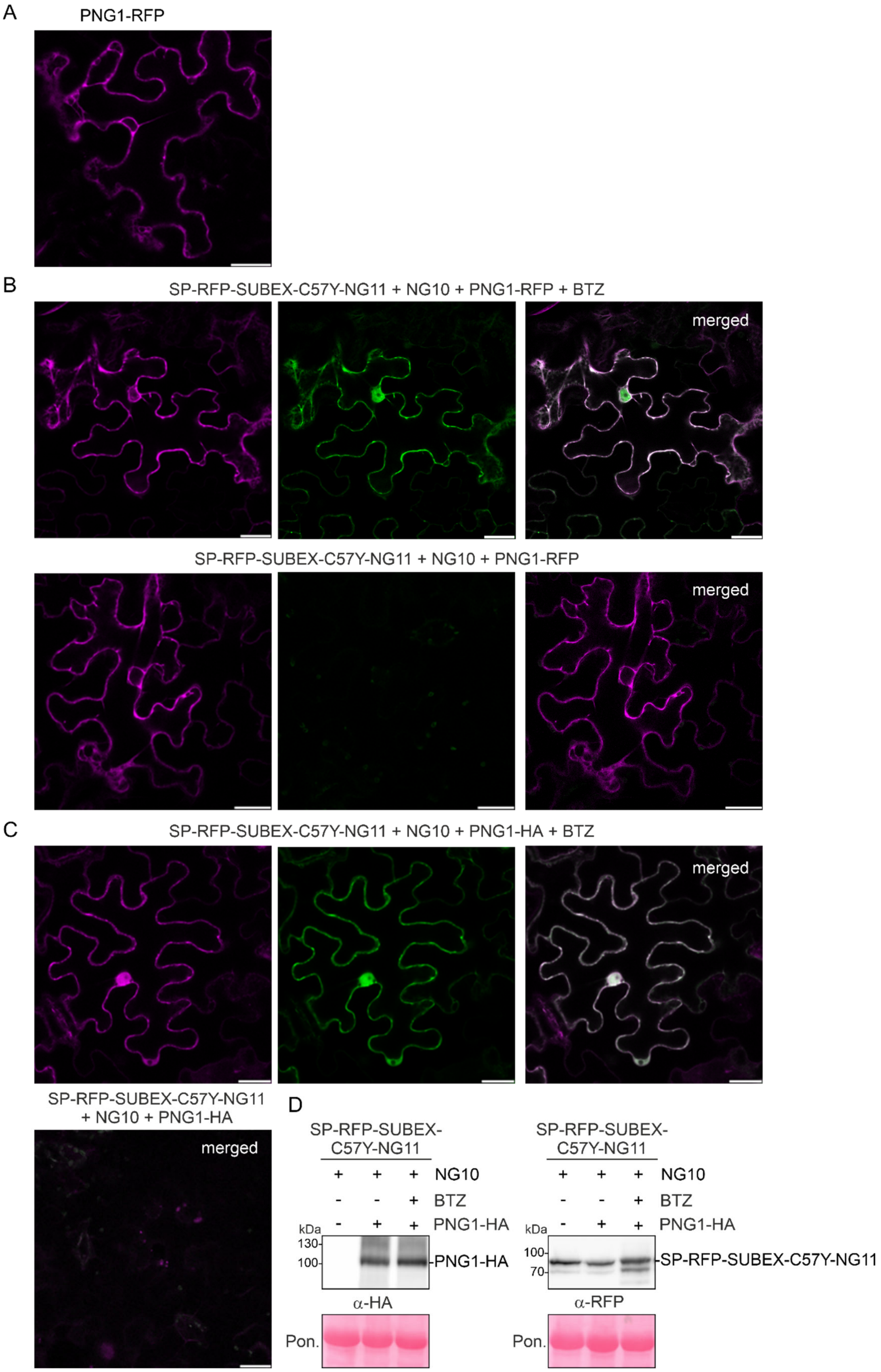
A
PNG1-RFP
B
SP-RFP-SUBEX-C57Y-NG11 + NG10 + PNG1-RFP + BTZ
merged
SP-RFP-SUBEX-C57Y-NG11 + NG10 + PNG1-RFP
merged
C
SP-RFP-SUBEX-C57Y-NG11 + NG10 + PNG1-HA + BTZ
merged
SP-RFP-SUBEX-C57Y-NG11
+ NG10 + PNG1-HA
merged
D
SP-RFP-SUBEX-C57Y-NG11
NG10
BTZ
PNG1-HA
kDa
130
100
PNG1-HA
α-HA
Pon.
SP-RFP-SUBEX-C57Y-NG11
NG10
BTZ
PNG1-HA
kDa
100
70
SP-RFP-SUBEX-C57Y-NG11
α-RFP
Pon.

Supplemental Figure S14. PNG1 co-expression does not lead to accumulation of the mNeonGreen2 signal in the cytosol or nucleus. (**A**) PNG1-RFP was transiently expressed in *N. benthamiana* leaf epidermal cells. A representative confocal image is shown. (**B**) SP-RFP-SUBEX-C57Y-NG11 was transiently co-expressed in *N. benthamiana* leaf epidermal cells with NG10 and PNG1-RFP in the presence or absence or of 20 μM BTZ. (**C**) SP-RFP-SUBEX-C57Y-NG11 was transiently co-expressed in *N. benthamiana* leaf epidermal cells with NG10 and PNG1-HA in the presence or absence of 20 μM BTZ. Scale bars = 10 μm. (**D**) Immunoblot analysis of SP-RFP-SUBEX-C57Y-NG11 transiently co-expressed in *N. benthamiana* leaves with NG10 and PNG1-HA.

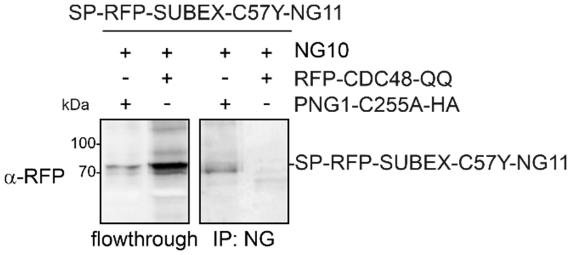

Supplemental Figure S15. No SP-RFP-SUBEX-C57Y-NG11 is purified with NG-trap beads when the degradation is blocked by RFP-CDC48-QQ co-expression. SP-RFP-SUBEX-C57Y-NG11 was transiently co-expressed in *N. benthamiana* leaves with NG10 and RFP-CDC48-QQ or PNG1-C255A-HA, purified by binding to NG-trap beads and the flowthrough as well as the purified fraction were analysed by immunoblotting. The absence of SP-RFP-SUBEX-C57Y-NG11 in the presence of NG10 and CDC48-QQ shows that the NG-trap beads are specific for reconstituted mNeonGreen2.

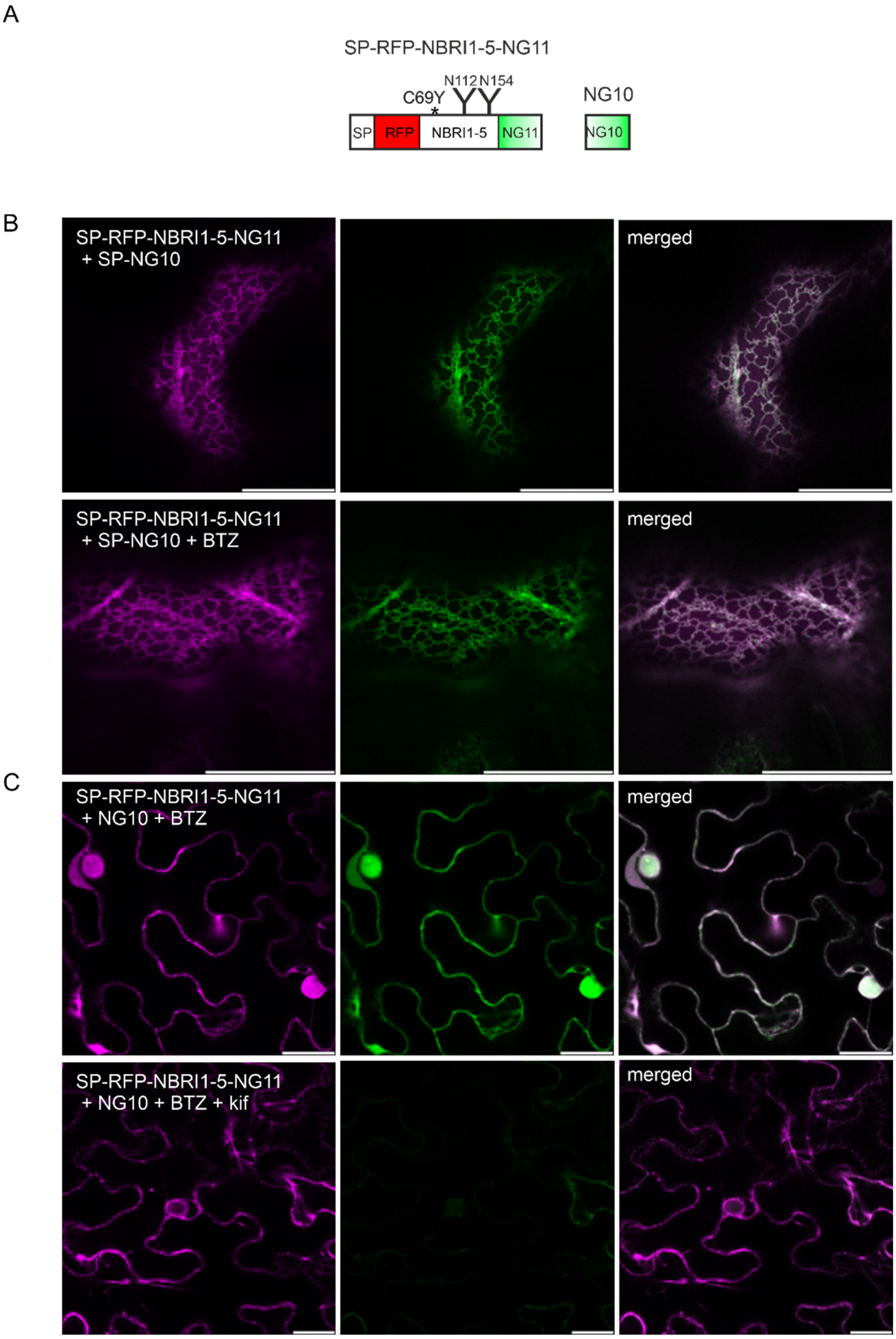

Supplemental Figure S16. The N-glycosylated ERAD substrate SP-RFP-NBRI1-5-NG11 is retrotranslocated and degraded by the proteasome. (**A**) Schematic illustration of the expressed protein. The C69Y amino acid exchange present in Arabidopsis *bri1-5* and the two N-terminal N-glycosylation (N112 and N154) sites of BRI1 are indicated (Shin et al., 2018). The numbering is according to the full-length *A. thaliana* BRI1 protein (UniProt: O22476). (**B** and **C**) Representative confocal images of SP-RFP-NBRI1-5-NG11 transiently co-expressed with SP-NG10 without or with 20 µM BTZ (**B**) or co-expressed in *N. benthamiana* leaf epidermal cells with NG10 in the presence of 20 µM BTZ or in the presence of 20 µM BTZ and 50 µM kif (**C**). Scale bars = 10 µm.

A

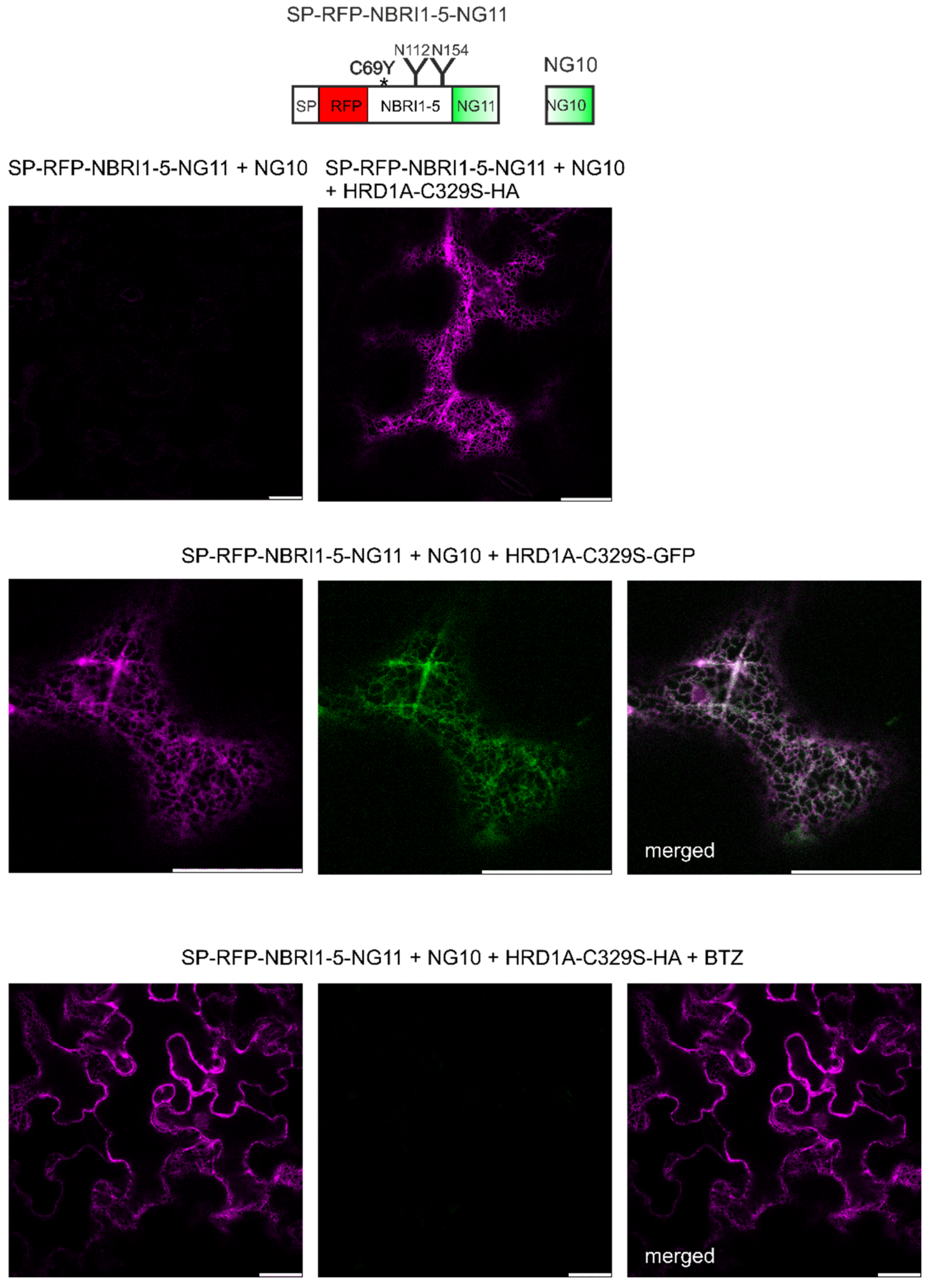

Figure S17. Retrotranslocation of the N-glycosylated ERAD substrate SP-RFP-NBRI1-5-NG11 is blocked by HRD1A-C329S co-expression. (**A**) Schematic illustration of the expressed protein. Representative confocal images of SP-RFP-NBRI1-5-NG11 transiently co-expressed with NG10 without or with HRD1A-C329S-HA, (**B**) or co-expressed in *N. benthamiana* leaf epidermal cells with NG10 and HRD1A-C329S-GFP, (**C**) or co-expressed in *N. benthamiana* leaf epidermal cells with NG10 and HRD1A-C329S-HA in the presence of 20 µM BTZ. Scale bars = 10 µm.

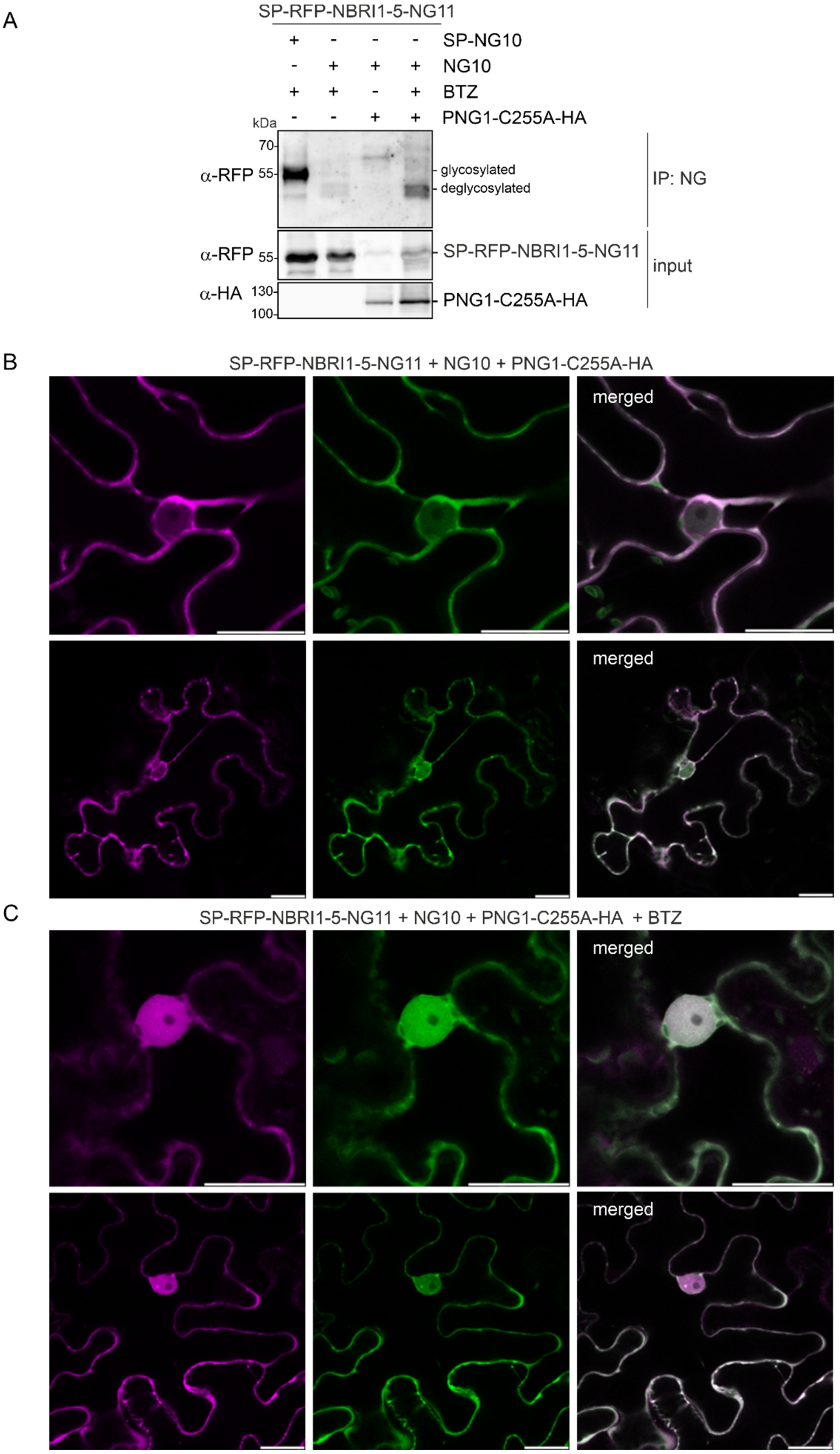
A
SP-RFP-NBRI1-5-NG11
SP-NG10
NG10
BTZ
PNG1-C255A-HA
kDa
70
55
α-RFP
glycosylated
deglycosylated
IP: NG
α-RFP
55
SP-RFP-NBRI1-5-NG11
input
α-HA
130
100
PNG1-C255A-HA
B
SP-RFP-NBRI1-5-NG11 + NG10 + PNG1-C255A-HA
merged
merged
C
SP-RFP-NBRI1-5-NG11 + NG10 + PNG1-C255A-HA + BTZ
merged
merged

Supplemental Figure S18. PNG1-C255A-HA blocks the degradation of the retrotranslocated N-glycosylated ERAD substrate SP-RFP-NBRI1-5-NG11. (**A**) Immunoblot analysis of NG-trap purified SP-RFP-NBRI1-5-NG11. The indicated proteins were transiently co-expressed in *N. benthamiana* leaves. (**B** and **C**) Representative confocal images of SP-RFP-NBRI1-5-NG11 transiently co-expressed in *N. benthamiana* leaf epidermal cells with NG10 and PNG1-C255A-HA in the absence (**B**) or presence (**C**) of 20 μM BTZ. Scale bars = 10 μm.

Table S1. List of synthetic DNA sequences used in this study.

name	sequence
NG10	ATGGTTTCTAAGGGCGAAGAGGACAACATGGCTTCTTTGCCTGCTACTCACGAGCTGCACATCTTCGGTTCTATCAACGGTGTGGAC<br>TTCGACATGGTTGGTCAAGGTACTGGCAACCCTAACGATGGTTACGAGGAACTGAACCTGAAGTCCACCAAGGGTGATCTGCAGTTC<br>TCTCCTTGGATTCTGGTGCCTCACATCGGTTACGGTTTCCACCAGTACCTGCCTTATCCTGATGGCATGTCTCCATTCCAGGCTGCT<br>ATGGTTGATGGCTCTGGTTATCAGGTGCACAGGACTATGCAGTTCGAGGATGGTGCTTCTCTCACCGTGAATTACAGGTACACCTAC<br>GAGGGCTCTCACATCAAGGGTGAAGCTCAGGTTATGGGTACTGGCTTTCCTGCTGATGGTCCTGTGATGACTAATACCCTTACCGCT<br>GCTGACTGGTGCATGAGCAAGAAAACTTACCCGAACGACAAGACCATCATCAGCACCTTCAAGTGGTCTTACACCACCGTGAACGGT<br>AAGAGGTACAGGTCTACTGCTAGGACCACTTACACCTTCGCTAAGCCTATGGCTGCCAACTACCTTAAGAACCAGCCGATGTACGTG<br>TTCCGTAAGACCGAGCTTAAGCACTCTATGTAG
SP-NG10	ATGGCTAACAAGCACCTGAGCCTGTCTCTGTTCCTTGTGCTTCTTGGTCTGTCTGCTTCTCTGGCTTCTGGATCCGTTTCTAAGGGC<br>GAAGAGGACAACATGGCTTCTTTGCCTGCTACTCACGAGCTGCACATCTTCGGTTCTATCAACGGTGTGGACTTCGACATGGTTGGT<br>CAAGGTACTGGCAACCCTAACGATGGTTACGAGGAACTGAACCTGAAGTCCACCAAGGGTGATCTGCAGTTCTCTCCTTGGATTCTG<br>GTGCCTCACATCGGTTACGGTTTCCACCAGTACCTGCCTTATCCTGATGGCATGTCTCCATTCCAGGCTGCTATGGTTGATGGCTCT<br>GGTTATCAGGTGCACAGGACTATGCAGTTCGAGGATGGTGCTTCTCTCACCGTGAATTACAGGTACACCTACGAGGGCTCTCACATC<br>AAGGGTGAAGCTCAGGTTATGGGTACTGGCTTTCCTGCTGATGGTCCTGTGATGACTAATACCCTTACCGCTGCTGACTGGTGCATG<br>AGCAAGAAAACTTACCCGAACGACAAGACCATCATCAGCACCTTCAAGTGGTCTTACACCACCGTGAACGGTAAGAGGTACAGGTCT<br>ACTGCTAGGACCACTTACACCTTCGCTAAGCCTATGGCTGCCAACTACCTTAAGAACCAGCCGATGTACGTGTTCCGTAAGACCGAG<br>CTTAAGCACTCTATGTAG
SUBEX-C57Y-NG11	ATGGTTACTAATCTACGAGATGTTTCGGCGATTAATAACTTGTATATCACTTTGGGAGCACCATCTCTACATCATTGGCTTGCTTTT<br>GGAGGAGACCCTTaTGGAGAAAAGTGGCAAGGTGTGGTGTGTGACTCCTCAAACATCACAGAAATAAGGATACCTGGCATGAAGGTA<br>GGTGGAGGCTTAAGTGATACTCTGGCTGATTTTTCATCTATCCAAGTCATGGACTTCAGTAGCAATCATATTTCAGGGACAATTCCG<br>CAGGCTTTGCCTTCTTCCATCCGAAACCTATCTCTCTCAAGCAATCGCTTCACTGGGAACATTCCCTTTACATTGTCCTTTCTATCC<br>GATTTGTCTGAACTGTCATTGGGAAGCAATCTTCTATCAGGAGAGATACCAGATTACTTTCAGCAGCTATCAAAACTGACAAAACTG<br>GACTTATCGTCTAACATACTGGAGGGGCATTTACCTTCTTCCATGGGAGACTTAGCTTCTCTTAAGATATTGTATTTGCAGGACAAC<br>AAGCTCACAGGAACACTTGATGTTATAGAGGATCTTTTCTTAACCGATTTGAATGTAGAAAACAACTTATTCTCGGGACCTATACCG<br>CCAAATCTATTGAAAATTCCAAACTTCAAAAAAGATGGAACTCCGTTCAATACATCGATTATAACACCACCGCCTCCGCCTGTGGTT<br>GATCCTCCTCCCGCTACTCACCGTGCTCCTCCTGTTCCCCGTATCCCTCCTGTTTCTGGTGTTCCCCCAGCACCTTTTGCTCCTTTT<br>GCTCCACTGCAACCACAACAACATCCACCACCATCACCACCTCTGGTCTGGTCACCACCTTCTTCTGATAATGGAGGAGGAGATCCC<br>TGGAACTCTGTGTCAGGGCAACCTACCTTGCAAATATCACCTCCTTCAGGTTCAGGATCAGGAAAATTCTGGTCCACTCAAAGAgga<br>tccGGCTCTGGCACCGAGCTCAACTTCAAGGAGTGGCAAAAGGCCTTTACCGATATGATGTAG
SP-SUBEX-C57Y-NG11	ATGAGCTTTACAAGATGGGAAGTGTTCTTTGGTCTCTCTGTTTTAGCCTTGACAATGCCTTTCTCAGCTGGAGTTACTAATCTACGA<br>GATGTTTCGGCGATTAATAACTTGTATATCACTTTGGGAGCACCATCTCTACATCATTGGCTTGCTTTTGGAGGAGACCCTTaTGGA<br>GAAAAGTGGCAAGGTGTGGTGTGTGACTCCTCAAACATCACAGAAATAAGGATACCTGGCATGAAGGTAGGTGGAGGCTTAAGTGAT<br>ACTCTGGCTGATTTTTCATCTATCCAAGTCATGGACTTCAGTAGCAATCATATTTCAGGGACAATTCCGCAGGCTTTGCCTTCTTCC<br>ATCCGAAACCTATCTCTCTCAAGCAATCGCTTCACTGGGAACATTCCCTTTACATTGTCCTTTCTATCCGATTTGTCTGAACTGTCA<br>TTGGGAAGCAATCTTCTATCAGGAGAGATACCAGATTACTTTCAGCAGCTATCAAAACTGACAAAACTGGACTTATCGTCTAACATA<br>CTGGAGGGGCATTTACCTTCTTCCATGGGAGACTTAGCTTCTCTTAAGATATTGTATTTGCAGGACAACAAGCTCACAGGAACACTT<br>GATGTTATAGAGGATCTTTTCTTAACCGATTTGAATGTAGAAAACAACTTATTCTCGGGACCTATACCGCCAAATCTATTGAAAATT<br>CCAAACTTCAAAAAAGATGGAACTCCGTTCAATACATCGATTATAACACCACCGCCTCCGCCTGTGGTTGATCCTCCTCCCGCTACT<br>CACCGTGCTCCTCCTGTTCCCCGTATCCCTCCTGTTTCTGGTGTTCCCCCAGCACCTTTTGCTCCTTTTGCTCCACTGCAACCACAA<br>CAACATCCACCACCATCACCACCTCTGGTCTGGTCACCACCTTCTTCTGATAATGGAGGAGGAGATCCCTGGAACTCTGTGTCAGGG<br>CAACCTACCTTGCAAATATCACCTCCTTCAGGTTCAGGATCAGGAAAATTCTGGTCCACTCAAAGAggatccGGCTCTGGCACCGAG<br>CTCAACTTCAAGGAGTGGCAAAAGGCCTTTACCGATATGATGTAG
p117-SP-SUBEX-C57Y-<br>NG11	GTTACTAATCTACGAGATGTTTCGGCGATTAATAACTTGTATATCACTTTGGGAGCACCATCTCTACATCATTGGCTTGCTTTTGGA<br>GGAGACCCTTaTGGAGAAAAGTGGCAAGGTGTGGTGTGTGACTCCTCAAACATCACAGAAATAAGGATACCTGGCATGAAGGTAGGT<br>GGAGGCTTAAGTGATACTCTGGCTGATTTTTCATCTATCCAAGTCATGGACTTCAGTAGCAATCATATTTCAGGGACAATTCCGCAG<br>GCTTTGCCTTCTTCCATCCGAAACCTATCTCTCTCAAGCAATCGCTTCACTGGGAACATTCCCTTTACATTGTCCTTTCTATCCGAT<br>TTGTCTGAACTGTCATTGGGAAGCAATCTTCTATCAGGAGAGATACCAGATTACTTTCAGCAGCTATCAAAACTGACAAAACTGGAC<br>TTATCGTCTAACATACTGGAGGGGCATTTACCTTCTTCCATGGGAGACTTAGCTTCTCTTAAGATATTGTATTTGCAGGACAACAAG<br>CTCACAGGAACACTTGATGTTATAGAGGATCTTTTCTTAACCGATTTGAATGTAGAAAACAACTTATTCTCGGGACCTATACCGCCA<br>AATCTATTGAAAATTCCAAACTTCAAAAAAGATGGAACTCCGTTCAATACATCGATTATAACACCACCGCCTCCGCCTGTGGTTGAT<br>CCTCCTCCCGCTACTCACCGTGCTCCTCCTGTTCCCCGTATCCCTCCTGTTTCTGGTGTTCCCCCAGCACCTTTTGCTCCTTTTGCT<br>CCACTGCAACCACAACAACATCCACCACCATCACCACCTCTGGTCTGGTCACCACCTTCTTCTGATAATGGAGGAGGAGATCCCTGG<br>AACTCTGTGTCAGGGCAACCTACCTTGCAAATATCACCTCCTTCAGGTTCAGGATCAGGAAAATTCTGGTCCACTCAAAGAggatcc<br>GGCTCTGGCACCGAGCTCAACTTCAAGGAGTGGCAAAAGGCCTTTACCGATATGATGTAG
p48-NG11-SUBEX-C57Y	ATGAGCTTTACAAGATGGGAAGTGTTCTTTGGTCTCTCTGTTTTAGCCTTGACAATGCCTTTCTCAGCTGGAACCGAGCTCAACTTC<br>AAGGAGTGGCAAAAGGCCTTTACCGATATGATGGgcAGATCTGTTACTAATCTACGAGATGTTTCGGCGATTAATAACTTGTATATC<br>ACTTTGGGAGCACCATCTCTACATCATTGGCTTGCTTTTGGAGGAGACCCTTaTGGAGAAAAGTGGCAAGGTGTGGTGTGTGACTCC<br>TCAAACATCACAGAAATAAGGATACCTGGCATGAAGGTAGGTGGAGGCTTAAGTGATACTCTGGCTGATTTTTCATCTATCCAAGTC<br>ATGGACTTCAGTAGCAATCATATTTCAGGGACAATTCCGCAGGCTTTGCCTTCTTCCATCCGAAACCTATCTCTCTCAAGCAATCGC<br>TTCACTGGGAACATTCCCTTTACATTGTCCTTTCTATCCGATTTGTCTGAACTGTCATTGGGAAGCAATCTTCTATCAGGAGAGATA<br>CCAGATTACTTTCAGCAGCTATCAAAACTGACAAAACTGGACTTATCGTCTAACATACTGGAGGGGCATTTACCTTCTTCCATGGGA<br>GACTTAGCTTCTCTTAAGATATTGTATTTGCAGGACAACAAGCTCACAGGAACACTTGATGTTATAGAGGATCTTTTCTTAACCGAT<br>TTGAATGTAGAAAACAACTTATTCTCGGGACCTATACCGCCAAATCTATTGAAAATTCCAAACTTCAAAAAAGATGGAACTCCGTTC<br>AATACATCGATTATAACACCACCGCCTCCGCCTGTGGTTGATCCTCCTCCCGCTACTCACCGTGCTCCTCCTGTTCCCCGTATCCCT<br>CCTGTTTCTGGTGTTCCCCCAGCACCTTTTGCTCCTTTTGCTCCACTGCAACCACAACAACATCCACCACCATCACCACCTCTGGTC<br>TGGTCACCACCTTCTTCTGATAATGGAGGAGGAGATCCCTGGAACTCTGTGTCAGGGCAACCTACCTTGCAAATATCACCTCCTTCA<br>GGTTCAGGATCAGGAAAATTCTGGTCCACTCAAAGA

OTU1_C92S	ATGCAGAATCAGATTGATATGGTGAAGGATGAAGCGGAAGTAGCTGCATCGATTTCAGCAATTAAGGGTGAAGAATGGGGAAATTGT<br>TCATCAGTGGAAGATCAACCATCTTTTCAAGAAGAAGAAGCTGCTAAAGTTCCTTATGTTGGTGATAAGGAACCTCTGTCTAGTTTA<br>GCTGCAGAGTATCAATCAGGGAGTCCCATTTTGCTGGAGAAGATTAAGATACTGGACAGTCAATATATCGGAATCCGGCGAACAAGA<br>GGAGATGGAAATaGCTTCTTCCGAAGTTTTATGTTCTCTTACCTTGAGCATATATTGGAATCACAAGATCGTGCTGAAGTCGATCGT<br>ATCAAGGTCAATGTTGAGAAATGTAGAAAGACTCTGCAAAACTTAGGTTATACAGATTTTACATTTGAGGACTTCTTTGCGTTGTTC<br>CTTGAGCAACTAGATGACATTCTCCAAGGAACTGAAGAGTCTATAAGCTACGATGAGCTGGTTAACAGAAGTAGAGATCAGTCAGTC<br>TCAGATTACATTGTAATGTTCTTTAGGTTTGTTACTGCTGGTGATATACGAACGCGTGCCGATTTTTTCGAGCCTTTTATAACAGGC<br>TTATCAAATGCAACAGTGGATCAGTTTTGCAAGTCCTCGGTCGAACCAATGGGGGAAGAGAGTGACCATATTCACATAACTGCTTTG<br>TCGGACGCACTTGGTGTTGCAATCCGTGTTGTGTATCTTGACCGTAGCTCATGTGATAGTGGGGGCGTCACTGTGAATCATCATGAC<br>TTTGTTCCTGTGGGCATTACCAATGAGAAAGATGAAGAAGCTTCTGCTCCATTTATAACCTTGCTGTATCGTCCAGGCCATTACGAT<br>ATCCTCTACCCCAAGCCATCTTGTAAGGTATCAGACAATGTGGGGAAA
OTU2_C16S	ATGGAAGGAATCATTGTGAGAAGAGTTATACCATCTGATAACAGTTCTCTCTTCAATGCAATCGGTTATGTCATGGACAAGGACAAA<br>AACAAAGCTCCTGAGCTCAGACAGGTGATAGCAGCAGCAGTTGCAAGCAACAAGGAGAAATATAATGAAGCATTTCTAGGGAAGCTC<br>AATGAAGAATATTGTGCTTGGATTCTCAATCCAGACAAGTGGGGAGGTGCGATTGAGCTTTCGATATTAGCAGATTATTATGGTCGA<br>GAAATTGCAGCTTACGACATTCAAACTAGTCGATGTGACTTGTATGGACAGACGAGAAACTACGACGAAAGAGTTATGCTGATCTAT<br>GACGGTCTTCATTACGATGCTCTTGCTCTGTCTCCATTTGAAGGGGCCGAGGAAGATTTTGATATGACTATATATCCAGTTGGTAAA<br>GATAGATCCATAGGATCAATTGAAGGGCTTGCTTTGAATCTAGTGAAGGACCAACAAAGGAAAAGGAGTTACACAGATACTGCAAAC<br>TTCACTCTACGTTGCGGTGTTTGCCAAATTGGAGTTATTGGACAAAAGGAAGCTGTGGAACATGCTCAAGCAACTGGTCATGTTAAT<br>TTTCAAGAATACAAA
PNG1_C255A	ATGGTGGCTAGGAAGTTCGCCGTTAGCCATAACGATAGCACCTTCGACATCGACTACGATACCGATGATGGTTTCGAGGTGCTGAAG<br>TACCAGCTGTTCTCTCTTACTTCCGTGCCTCCTGATCAGCAAAAGATCCTTGGTAGGGATGACCAGATCGTGAGCGACGAATCTGAT<br>CTGGCTAGCATCTCTGATAAGCTGAGGCTGGTGTCTCTGGATGGTGATGCTGATGAGATCAGCGCCCAAGAGAAAGAGAAGTCCGAG<br>ATCGCTATGTCCGATGAGGAACTGGCTAGGATTCTGCAAGCTGAAGAAGAGGCTCTGATGATGCAGCAGTTCGTGGCCTCTGAAAAC<br>AAAGAGCAGGTTGAGCAGCGGATCAGGCCGTATGTTAATCAGGTGCTCATGTACGAGGACCTGCACAGACAAGAGATGGCTAGAAAG<br>ACTGTGCCTGTGGACAAGCTGGAAGAGAAGGCTTTGATCTCTCTGGGTAGAGAGGGTAACCTGAGGCCTTCTAAGGTGGAACAGGAT<br>AACGCTTTCCTGCTCCAGCTTCTGTTCTGGTTCAAGCAGTCTTTCCGGTGGGTTAACGCTCCTCCTTGTGATTCTTGCGGTAACGAG<br>ACTAGGTCCCAAGGTATGGGTGTCGCTAACTCTTCTGAGACTCAGTACGGTGCTTCTCGGGTTGAGCTTTACAGGTGCAACAGCTGC<br>TCCAACATCACTAGGTTCCCTAGGTACAACGACCCTCTGAAGCTTCTCGAGACTAGAAAGGGTAGAGCTGGTGAGTGGGCTAACTGC<br>TTCACTCTTTACTGCAAGGCTTTCGGCTACGACTCTAGGCTGATTCTGGATTTCACTGACCATGTGTGGACCGAGTGCTTCTCACCT<br>TCACTTGGTAGATGGATGCACCTCGATCCTTGCGAGGGAATCTACGATAACCCTCTGCTGTACGAGAAAGGCTGGAAGAAAAACCTG<br>AACTACGTGATCGCTATCGCCAAGGATGGTGTGCACGATGTGACTAAGAGGTACACTAGGAAGTGGCCCGAGGTTTTGTCTCGGAGG<br>AATATTACTTCTGAGCCCGCTCTTTCTGCCGTGCTGTCTGATATTACTCGTGAGCTTCAGAAGAAGCTGAGCGCCGAAGTTATCAGC<br>GCTCTTGAGGATAGAGATCGGACCGAGATGGATGTGATTGAGCGTGAGCTGTACTCCAAGGATGATGCTGTTGTGTCTCTGCCTGGT<br>AGGCTGTCTGGTGACAAAGAATGGCGTATTGCCAGGTCCGAGTTCGTGTCTGATGAGAAGAACAGCCTGTCCAGCTCTTTTTGCCCT<br>GTGAGAAGATGCGTGGACGATCACGTGACCAAGATATACAGCGCTTTCAGCCCTGTGCTGACCAAGCTTATTGAGTACAGCCCTTCT<br>AAGACCACCGCTATCCAGGTGCTCGAGATTTTCCGGAAGATTCTGGTGGACCTGAAGTCCTCTCCATTCAGGACTAGAAGGACCAGC<br>GTGAAGTCCGTGTCTAGTAGCTCTGGGGAGATTTTCTCTAAGACCCTGGCTTCTTTCGGCCAGCTTCTTGATGCTCTGTCTCTGAAG<br>TCTGAGCTGGGTAGCAACGGCAGCATCGATATTTCTCTGGCTTCCGATCCGGTCAAGACCTCTGTTGCTTTGCCTGTTGTTTTCCAC<br>GCTGTGGACGACGTGATCTACAATGTTGGTCAGTGCGCTAGGCTGGACTCTCGTTCTTTGGCTTGGCCTCTTTTGAAGCTGAACAGG<br>CTTTGCTCTGGTCTGGTGCTTGCTTCTGGTGAGGAACTTCCTTTCGGTATTGCTACCTGCGCTTTCGATGGAACCAGGATGTCTAAG<br>TGGGAAGAACCTAATGGTGCTGCTGGCTGCTGGATCATCTATCAGGTTGCAGGTAACCGGATGTTCGAGCTTGTTGCTTACGAGCTG<br>ATGAGCGCTAATGATGCTCCTGAGAGAGATCCTAAGGACTGGGTTCTCGAGGGTTCTGAGAATGGTGGTTCTTCTTGGCACCTCCTG<br>GATAAGCAGACCAGCCAGATGTTCGATAAGCGGTTCCAGCGTAAGACCTTCACCGTTTCTTCACCTGGTTACCTGGCTAACGCATTC<br>CGGCTTAGATTCCTTGCTGTGAGGGATGCTAACGCCAACTCTAGGTTCCAGATCGGCTCTATCGATCTGTACGCCTCCTCTTCT
PNG1	ATGGTGGCTAGGAAGTTCGCCGTTAGCCATAACGATAGCACCTTCGACATCGACTACGATACCGATGATGGTTTCGAGGTGCTGAAG<br>TACCAGCTGTTCTCTCTTACTTCCGTGCCTCCTGATCAGCAAAAGATCCTTGGTAGGGATGACCAGATCGTGAGCGACGAATCTGAT<br>CTGGCTAGCATCTCTGATAAGCTGAGGCTGGTGTCTCTGGATGGTGATGCTGATGAGATCAGCGCCCAAGAGAAAGAGAAGTCCGAG<br>ATCGCTATGTCCGATGAGGAACTGGCTAGGATTCTGCAAGCTGAAGAAGAGGCTCTGATGATGCAGCAGTTCGTGGCCTCTGAAAAC<br>AAAGAGCAGGTTGAGCAGCGGATCAGGCCGTATGTTAATCAGGTGCTCATGTACGAGGACCTGCACAGACAAGAGATGGCTAGAAAG<br>ACTGTGCCTGTGGACAAGCTGGAAGAGAAGGCTTTGATCTCTCTGGGTAGAGAGGGTAACCTGAGGCCTTCTAAGGTGGAACAGGAT<br>AACGCTTTCCTGCTCCAGCTTCTGTTCTGGTTCAAGCAGTCTTTCCGGTGGGTTAACGCTCCTCCTTGTGATTCTTGCGGTAACGAG<br>ACTAGGTCCCAAGGTATGGGTGTCGCTAACTCTTCTGAGACTCAGTACGGTGCTTCTCGGGTTGAGCTTTACAGGTGCAACAGCTGC<br>TCCAACATCACTAGGTTCCCTAGGTACAACGACCCTCTGAAGCTTCTCGAGACTAGAAAGGGTAGAtgTGGTGAGTGGGCTAACTGC<br>TTCACTCTTTACTGCAAGGCTTTCGGCTACGACTCTAGGCTGATTCTGGATTTCACTGACCATGTGTGGACCGAGTGCTTCTCACCT<br>TCACTTGGTAGATGGATGCACCTCGATCCTTGCGAGGGAATCTACGATAACCCTCTGCTGTACGAGAAAGGCTGGAAGAAAAACCTG<br>AACTACGTGATCGCTATCGCCAAGGATGGTGTGCACGATGTGACTAAGAGGTACACTAGGAAGTGGCCCGAGGTTTTGTCTCGGAGG<br>AATATTACTTCTGAGCCCGCTCTTTCTGCCGTGCTGTCTGATATTACTCGTGAGCTTCAGAAGAAGCTGAGCGCCGAAGTTATCAGC<br>GCTCTTGAGGATAGAGATCGGACCGAGATGGATGTGATTGAGCGTGAGCTGTACTCCAAGGATGATGCTGTTGTGTCTCTGCCTGGT<br>AGGCTGTCTGGTGACAAAGAATGGCGTATTGCCAGGTCCGAGTTCGTGTCTGATGAGAAGAACAGCCTGTCCAGCTCTTTTTGCCCT<br>GTGAGAAGATGCGTGGACGATCACGTGACCAAGATATACAGCGCTTTCAGCCCTGTGCTGACCAAGCTTATTGAGTACAGCCCTTCT<br>AAGACCACCGCTATCCAGGTGCTCGAGATTTTCCGGAAGATTCTGGTGGACCTGAAGTCCTCTCCATTCAGGACTAGAAGGACCAGC<br>GTGAAGTCCGTGTCTAGTAGCTCTGGGGAGATTTTCTCTAAGACCCTGGCTTCTTTCGGCCAGCTTCTTGATGCTCTGTCTCTGAAG<br>TCTGAGCTGGGTAGCAACGGCAGCATCGATATTTCTCTGGCTTCCGATCCGGTCAAGACCTCTGTTGCTTTGCCTGTTGTTTTCCAC<br>GCTGTGGACGACGTGATCTACAATGTTGGTCAGTGCGCTAGGCTGGACTCTCGTTCTTTGGCTTGGCCTCTTTTGAAGCTGAACAGG<br>CTTTGCTCTGGTCTGGTGCTTGCTTCTGGTGAGGAACTTCCTTTCGGTATTGCTACCTGCGCTTTCGATGGAACCAGGATGTCTAAG<br>TGGGAAGAACCTAATGGTGCTGCTGGCTGCTGGATCATCTATCAGGTTGCAGGTAACCGGATGTTCGAGCTTGTTGCTTACGAGCTG<br>ATGAGCGCTAATGATGCTCCTGAGAGAGATCCTAAGGACTGGGTTCTCGAGGGTTCTGAGAATGGTGGTTCTTCTTGGCACCTCCTG<br>GATAAGCAGACCAGCCAGATGTTCGATAAGCGGTTCCAGCGTAAGACCTTCACCGTTTCTTCACCTGGTTACCTGGCTAACGCATTC<br>CGGCTTAGATTCCTTGCTGTGAGGGATGCTAACGCCAACTCTAGGTTCCAGATCGGCTCTATCGATCTGTACGCCTCCTCTTCT

Table S2. List of primers used in this study.

name	sequence
SUB_F	TATATCTAGAGTTACTAATCTACGAGATGTTTC
SUB_R	TATAAGATCTTCTTTGAGTGGACCAGAATTTTCC
At1g18260_F	TATAACTAGTCGTCCCGTCGTTCTCGTCCTCTCA
At1g18260_R	TATAACTAGTTTACCGTGGGAACGCAGCGAGGTG
At3g16090_F	TTCTAGAATGATTCGACTAAGAACATACGCA
At3g16090_R	TGGATCCCTCTGCTGCATCAGCAACCGACT
At3g16090_MF	CAAGTGCTCTGCTAGTAGGACATGTCTGCTGTCTCT
At3g16090_MR	AGAGACAGCAGACATGTCCTACTAGCAGAGCACTTG
At5g35080_F	TATATCTAGACAGATCTTCCCAGCTCATCTAGTTG
At5g35080_R	TATAGGATCCTCAAGAATCAGCTATCATCTTAGGT
OTU1_F	TATATCTAGACAGAATCAGATTGATATGGTG
OTU1_R	TATAAGATCTTCATTTCCCCACATTGTCTGATAC
OTU1_XF	TATATCTAGAATGCAGAATCAGATTGATATGGTG
OTU1_XR	TATAAGATCTTTTCCCCACATTGTCTGATACC
OTU2_F	TATATCTAGAGAAGGAATCATTGTGAGAAGAG
OTU2_R	TATAGGATCCTTATTTGTATTCTTGAAAATTAAC
OTU2_MF	TTCTAGGGAAGCTCAATGAAGAATATAGTGCTTGGATTCTC
OTU2_MR	GAGAATCCAAGCACTATATTCTTCATTGAGCTTCCCTAGAA
BRI1_F	TATAACTAGTTTTCAAGCTTCACCATCTCAGTCT
BRI1_R	TATAGGATCCGATTTTGTTTCCGCTAATCGCTAA
